# The Hexosamine Biosynthesis Pathway: Regulation and Function

**DOI:** 10.3390/genes14040933

**Published:** 2023-04-18

**Authors:** Alysta Paneque, Harvey Fortus, Julia Zheng, Guy Werlen, Estela Jacinto

**Affiliations:** Department of Biochemistry and Molecular Biology, Robert Wood Johnson Medical School, Rutgers, The State University of New Jersey, Piscataway, NJ 08854, USA; amp536@rwjms.rutgers.edu (A.P.); hlf29@scarletmail.rutgers.edu (H.F.); jlz56@scarletmail.rutgers.edu (J.Z.); guy.werlen@rutgers.edu (G.W.)

**Keywords:** hexosamine biosynthesis pathway, GFAT1, GFAT2, Gfpt1, glycosylation, metabolism, UDP-GlcNAc, *O*-GlcNAcylation, glucosamine, protein folding

## Abstract

The hexosamine biosynthesis pathway (HBP) produces uridine diphosphate-*N*-acetyl glucosamine, UDP-GlcNAc, which is a key metabolite that is used for *N*- or *O*-linked glycosylation, a co- or post-translational modification, respectively, that modulates protein activity and expression. The production of hexosamines can occur via de novo or salvage mechanisms that are catalyzed by metabolic enzymes. Nutrients including glutamine, glucose, acetyl-CoA, and UTP are utilized by the HBP. Together with availability of these nutrients, signaling molecules that respond to environmental signals, such as mTOR, AMPK, and stress-regulated transcription factors, modulate the HBP. This review discusses the regulation of GFAT, the key enzyme of the de novo HBP, as well as other metabolic enzymes that catalyze the reactions to produce UDP-GlcNAc. We also examine the contribution of the salvage mechanisms in the HBP and how dietary supplementation of the salvage metabolites glucosamine and *N*-acetylglucosamine could reprogram metabolism and have therapeutic potential. We elaborate on how UDP-GlcNAc is utilized for *N*-glycosylation of membrane and secretory proteins and how the HBP is reprogrammed during nutrient fluctuations to maintain proteostasis. We also consider how *O*-GlcNAcylation is coupled to nutrient availability and how this modification modulates cell signaling. We summarize how deregulation of protein *N*-glycosylation and *O*-GlcNAcylation can lead to diseases including cancer, diabetes, immunodeficiencies, and congenital disorders of glycosylation. We review the current pharmacological strategies to inhibit GFAT and other enzymes involved in the HBP or glycosylation and how engineered prodrugs could have better therapeutic efficacy for the treatment of diseases related to HBP deregulation.

## 1. Introduction

**Figure 1:**
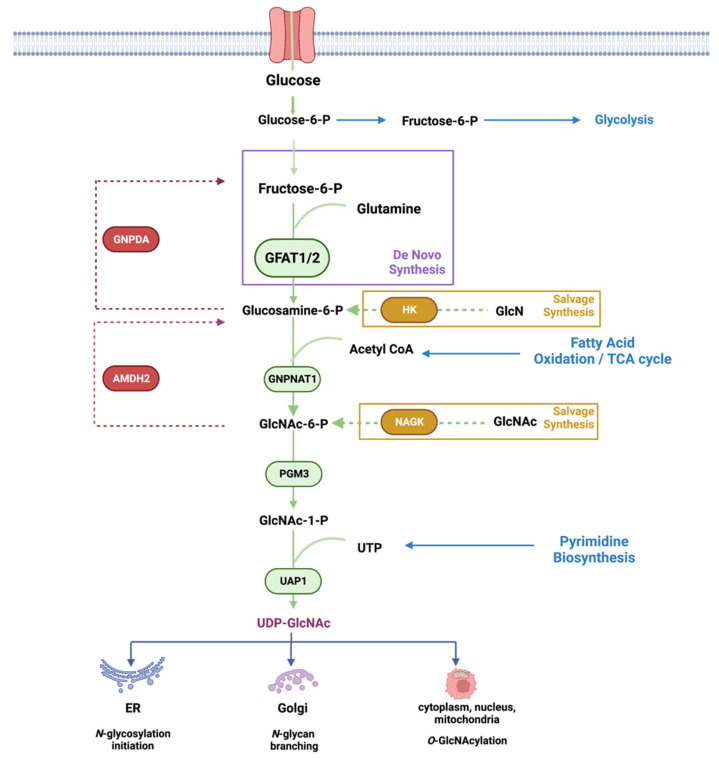
The hexosamine biosynthesis pathway. The de novo synthesis of UDP-GlcNAc is catalyzed by GFAT1/2, which utilizes glutamine and fructose-6-phosphate (a glycolytic metabolite) as substrates to generate glucosamine-6-phosphate. Subsequent reactions are catalyzed by metabolic enzymes to eventually generate UDP-GlcNAc, a metabolite that is used for protein *N*-glycosylation, *N*-glycan branching, and *O*-GlcNAcylation. UDP-GlcNAc can also be generated by using the salvage nutrients glucosamine (GlcN) and *N*-acetylglucosamine (GlcNAc).

Metabolic pathways are driven by the availability of nutrients and other environmental signals such as growth factors and stress stimuli. These pathways are critical for the production of energy and the synthesis of macromolecules including carbohydrates, lipids, amino acids, and nucleotides. Metabolic intermediates are also utilized for the modification of macromolecules. The nucleotide ATP is used for protein phosphorylation, a modification that alters protein conformation, activity, and function. Hexosamines, which are used for glycosylation, also serve to modify proteins, lipids, and nucleic acids [1,2,3]. The glycosylation of macromolecules can modulate their activity, stability, and function. The hexosamine biosynthesis pathway (HBP) generates a key metabolite, uridine-5′-di-phospho-*N*-acetylglucosamine (UDP-GlcNAc), which is utilized as the substrate for asparagine (*N*)-linked glycosylation of secretory and cell-surface proteins (Figure 1). It is also used for *O*-GlcNAcylation of intracellular proteins at serine/threonine residues. UDP-GlcNAc synthesis relies on the presence of carbohydrates (which serves as a carbon source), glutamine (nitrogen source), acetyl CoA (for acetylation), and UTP (energy source). UDP-GlcNAc can be generated de novo by using glucose and glutamine as the main nutrients. It can also be generated via salvage pathways that utilize glucosamine (GlcN) or *N*-acetylglucosamine (GlcNAc) as substrates. In addition, UDP-GlcNAc can be reversibly interconverted to UDP-GalNAc, and both of these metabolites are termed UDP-HexNAc [4].

*N*-glycosylation occurs in the endoplasmic reticulum (ER), wherein a high mannose glycan is preassembled onto two successive UDP-GlcNAc moieties that are attached to a dolichol embedded in the ER membrane. This precursor oligosaccharide is then transferred *en bloc* to Asn residues within Asn-X-Ser/Thr motifs of nascent secretory or membrane-bound polypeptides. UDP-GlcNAc is further used for *N*-glycan remodeling or branching of glycoproteins that undergo maturation in the Golgi. *N*-glycosylation of membrane and secretory proteins promotes their proper folding and enhances their stability. Hence, defects in *N*-glycosylation produce misfolded proteins, generate ER stress, remodel the proteome at the cell surface, and, consequently, affect cell signaling and cell fate [5,6]. UDP-GlcNAc is also a substrate for the *O*-GlcNAcylation of intracellular proteins on their serine/threonine residues. *O*-GlcNAcylation occurs in signaling proteins, transcription factors, and metabolic regulators. Hence, defects in *O*-GlcNAcylation also impact signaling, metabolism, and cell fate. 

This review will discuss the regulation of key enzymes of the de novo and the salvage HBP in eukaryotes, particularly in mammals. For a discussion on prokaryotic HBP enzymes, we refer the reader to a recent excellent review [7]. We also provide an overview of the functions of the HBP and how its deregulation could lead to diseases such as cancer, diabetes, immunodeficiencies, and congenital growth disorders. Finally, we summarize current therapeutic strategies to manipulate the HBP to prevent pathological conditions. 

## 2. De Novo Hexosamine Biosynthesis

Several key nutrients or metabolites that are acquired either from the environment or from intracellular sources enter the HBP. Glucose, which undergoes glycolysis to generate ATP, also generates other intermediates including fructose-6-phosphate (fructose-6-P), which is used for the de novo production of UDP-GlcNAc, the end product of the HBP. Approximately 2–3% of total glucose uptake is estimated to be utilized by the HBP in cultured adipocytes, but it is not entirely clear how much glucose fluxes through this pathway under different growth conditions or in distinct tissues [8]. In addition to fructose-6-P, de novo HBP also requires glutamine, which is acquired either extracellularly or intracellularly. However, flux through de novo HBP, which is catalyzed by GFAT, is not entirely dependent on substrate levels. In fact, some studies indicate that increased aerobic glycolysis and glutaminolysis limit de novo UDP-GlcNAc biosynthesis in T cell blasts [9]. In cultured adipocytes, increased glucose exposure for 3 hr enhances UDP-GlcNAc levels by about 2-fold, but no further increase occurs with higher glucose concentrations [10,11]. Acute glucose limitation in HeLa cells does not alter UDP-GlcNAc levels significantly either [12]. Furthermore, recent ex vivo studies using mouse heart indicate that acute changes in glucose levels or cardiac workload do not dramatically alter glucose flux through the HBP, despite increased glycolysis [13]. Studies that have used total cellular *O*-GlcNAcylation as a readout for HBP flux also suggest that glucose limitation or glycolytic inhibition may enhance UDP-GlcNAc production [12]. Glutamine limitation suppresses de novo HBP but triggers the salvage pathway to maintain UDP-GlcNAc abundance in pancreatic cancer [14]. Thus, the HBP (whether by de novo or salvage mechanisms) maintains UDP-GlcNAc levels tightly in response to nutrient fluctuations in order to establish cellular homeostasis. Accumulating evidence demonstrates that the expression and activity of GFAT could critically regulate flux through the de novo HBP. Feedback inhibition of GFAT1 and other enzymes along the HBP by metabolic products also accounts for the stringent regulation of this pathway. Furthermore, the requirement for and/or activation of de novo synthesis would be dependent on the biosynthetic and metabolic needs of distinct cell types. 

Whereas extracellular signals from the environment have input into the HBP, cell signaling pathways control the uptake of nutrients. Among these signals, insulin, which triggers the PI3K/mTOR intracellular signaling pathway, mediates the uptake and metabolism of glucose [15]. Depending on cell types, other growth factors that also activate the PI3K/mTOR pathway induce the uptake and metabolism of glucose as well as glutamine [16]. In addition to growth signals, environmental or nutrient stress is also relayed to the HBP [5,17]. Thus, excessive or limited glucose levels, stress of the endoplasmic reticulum (ER), or glutamine limitation all trigger the integrated stress response to modulate flux through the HBP, which eventually restores cellular homeostasis. We will discuss how the key metabolic enzymes that catalyze the reactions along the HBP are regulated by metabolites and signaling molecules, which serve to control the flux through this pathway. 

### 2.1. GFAT

The key enzyme in the de novo production of UDP-GlcNAc through the HBP is glutamine: fructose-6-phosphate amidotransferase (GFAT or GFPT). GFAT is present in all cellular organisms, including prokaryotes and eukaryotes, underscoring the importance of this enzyme and the HBP for normal cell function. The two mammalian paralogs, GFAT1 (GFPT1) and GFAT2 (GFPT2), share 75–80% amino acid sequence identity [18]. GFAT1 is found more ubiquitously, whereas GFAT2 seems to have higher expression in tissues of the central nervous system. Different cell types within a tissue may also have preference for the expression of either GFAT1 or GFAT2. For example, whereas GFAT1 is the primary isoform in cardiac myocytes, GFAT2 seems to predominate in cardiac fibroblasts [19]. In humans, an alternatively spliced form of hGFAT1 termed GFAT1-L or GFAT1Alt is highly expressed exclusively in striated muscle [20,21]. As discussed below, while GFAT1 and GFAT2 share mostly overlapping control mechanisms, they also have some different modes of regulation; hence, each could distinctly modulate de novo HBP. Tissue specificity and/or unique regulatory mechanisms of each of these paralogs remain to be further investigated. 

**Figure 2:**
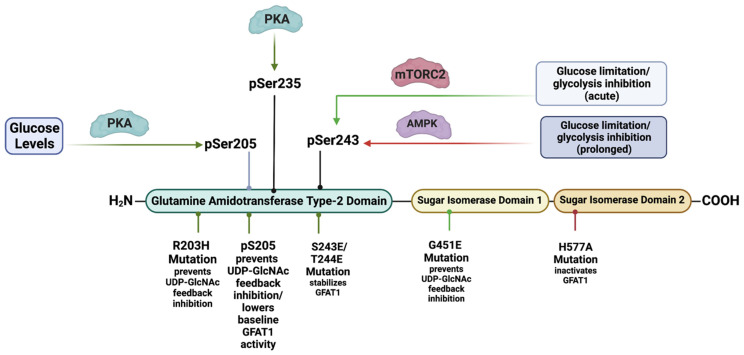
GFAT structural domains, regulatory sites, and protein kinase regulators. Green arrows indicate positive regulation, while the red arrow depicts negative regulation.

GFAT comprises an N-terminal 27 kDa glutaminase domain that catalyzes the hydrolysis of glutamine to glutamate and ammonia (Figure 2). The C-terminus harbors the 40 kDa isomerase domains that use ammonia to convert fructose-6-P (a glycolytic intermediate) to glucosamine-6-phosphate (GlcN-6-P) [22]. This reaction is the rate-limiting step of the de novo synthesis of hexosamines. Structural studies in *E. coli* reveal that GlmS (the GFAT counterpart) occur as functional dimers and that the glutamine and isomerase domains are linked with a solvent-inaccessible hydrophobic channel that transfers ammonia between the two domains [23]. GFAT is controlled at different levels including allosteric regulation by metabolites, post-translational modifications, and the modulation of mRNA and protein expression. Such regulation ensures that de novo hexosamine biosynthesis is attuned with other metabolic pathways in response to nutrient availability and the presence of appropriate environmental and cellular signals. 

HBP substrates and metabolites allosterically modulate the activity of GFAT. The binding of fructose-6-P to the isomerase domain of GFAT repositions the flexible glutaminase domain and subsequently promotes the association of glutamine (Gln) to the latter [24]. Upon Gln binding, the hydrophobic channel that connects the substrate-binding sites of the enzyme opens and allows the transfer of ammonia to generate GlcN-6-phosphate (GlcN-6-P) [23]. The production of GlcN-6-P inhibits the enzymatic activity of GFAT1 by competing with fructose-6-P binding to GFAT1 [25]. However, while GlcN-6-P can inhibit the transfer reaction of ammonia to fructose-6-P, the glutaminase activity of GFAT1 remains constant. Furthermore, the HBP end product known as UDP-GlcNAc also binds to GFAT1 to negatively regulate its activity [26]. GFAT1 gain-of-function mutations (such as G451E) expressed in both *Caenorhabditis elegans* and mouse neuroblastoma cells prevent its feedback inhibition via UDP-GlcNAc [26].

The phosphorylation of GFAT1 at Ser205, Ser235, and Ser243 also modulates its activity. PKA mediates the Ser205 phosphorylation, which is present in both GFAT1 and GFAT2 (homologous Ser202 in the latter) [27]. Notably, cell treatment with the PKA agonist known as forskolin either increases [28] or decreases [29] the in vitro activity of GFAT1. Tissue-specific effects or differences in assay conditions may explain the discrepancies in these findings. However, more recent studies report that PKA-mediated Ser205 phosphorylation stabilizes the glutaminase and isomerase domains of GFAT1 while preventing the UDP-GlcNAc feedback inhibition, which results in augmented enzymatic activity [30]. Furthermore, a gain-of-function mutation of GFAT1, R203H, which confers resistance to tunicamycin-induced proteotoxic stress, also increases the activity of the mutant while precluding the feedback inhibition of UDP-GlcNAc. Interestingly, PKA can no longer phosphorylate the R203H mutant at Ser205. It is thus possible that both the R203H mutation and the PKA-mediated Ser205 phosphorylation of wild-type GFAT1 induce a conformational shift that prevents the inhibitory binding of UDP-GlcNAC. The PKA-mediated phosphorylation of GFAT1 at Ser205 would, therefore, uncouple the UDP-GlcNAc feedback loop. Unleashing the activity of GFAT1 by relieving the UDP-GlcNAc-mediated feedback inhibition would facilitate and increase the flux through de novo HBP when PKA signals are elevated, despite scarce substrate levels for GFAT1. Indeed, PKA is also involved in modulating glucose homeostasis, primarily by antagonizing glycolysis (while promoting gluconeogenesis in the liver) [31]. Thus, maintaining GFAT1 activity via the phosphorylation of Ser205 could allow UDP-GlcNAc synthesis despite limited levels of the glycolysis metabolite fructose-6-P. Although no direct evidence has been documented so far, PKA may also mediate the phosphorylation of GFAT1 at Ser235 in addition to that at Ser205. The phosphorylation at Ser235 does not change the activity of GFAT1 in vitro [29]. Furthermore, GFAT2 is not phosphorylated at Ser235. Hence, the function of this phosphorylation in GFAT1 remains unclear. How PKA mediates GFAT1 activity in different tissues and in response to different stimuli warrants further investigation.

GFAT1 is also phosphorylated at Ser243. The phosphorylation of this site is linked to increased glucose limitation and glycolysis inhibition [12,32,33,34]. Previous studies demonstrate that the phosphorylation of this site is mediated by AMPK [32]. This is also consistent with findings that the activation of AMPK, such as via pharmacological means or via glucose starvation, correlates with increased phosphorylation at Ser243. Increased Ser243 phosphorylation has been proposed to diminish GFAT1 activity [32]. In support of this notion, AMPK agonists reduce GFAT1 activity in vitro. However, such analysis should be taken with caution since the levels of metabolites, in particular UDP-GlcNAc, could allosterically inhibit GFAT1 activity in vitro. Decreased GFAT1 activity upon AMPK upregulation in endothelial cells also correlates with diminished total *O*-GlcNAcylation of cellular proteins, implying diminished flux through the HBP [33]. Together, these findings are in line with a negative regulatory role of Ser243 phosphorylation in modulating GFAT1 activity. However, other signaling molecules besides AMPK might phosphorylate Ser243, and thus, the role of AMPK in regulating GFAT1 activity as well as promoting flux through the HBP requires further scrutiny. Notably, basal levels of GFAT1 phosphorylated at Ser243 are present in MEFs despite the abrogation of AMPK [33]. Furthermore, the amplitude and duration of Ser243 phosphorylation is modulated in an mTORC2-dependent manner when highly proliferating cancer cells are subjected to either glucose or glutamine-limited conditions [12]. On one hand, the degree or amplitude of Ser243 phosphorylation is high during glucose starvation and glycolysis inhibition. On the other hand, the amplitude and duration of this phosphorylation decline as glutamine levels diminish, and the maintenance of Ser243 phosphorylation under both glucose- and glutamine-limited conditions is dependent on the presence of mTORC2. Indeed, the phosphorylation of Ser243, the activation of mTORC2, and the amount of total *O*-GlcNAcylation correlate during glucose or combined glucose/glutamine-starved conditions. In contrast, AMPK activation correlates with Ser243 phosphorylation only during prolonged glucose starvation. Importantly, UDP-GlcNAc levels in HeLa cells are comparable in glucose- or combined glucose/glutamine-replete or -deplete conditions, indicating that flux through the HBP is maintained under acute nutrient-limited conditions [12]. However, GFAT1 expression and Ser243 phosphorylation decline as glutamine is depleted. Interestingly, phosphomimetic mutations of Ser243 and the adjacent Thr244 enhance GFAT1 expression and stability during nutrient starvation. Based on these findings, mTORC2-mediated GFAT1 Ser243 phosphorylation could serve to modulate flux through the HBP in conjunction with the availability of glucose and glutamine. Corroborating this supposition, Ser243 phosphorylation (along with mTORC2 activation) becomes more robust as mouse T cell lymphoma develops during PTEN deficiency [12]. Lymphomagenesis is characterized by increased demand for nutrients, thus mimicking a state of nutrient limitation. Thus, when nutrients are acutely limited or cellular demand is high, the HBP would need to be tightly regulated to restore metabolic homeostasis, arguing for a positive regulatory role for GFAT1 Ser243 phosphorylation. The RNA-binding protein AUF1, which controls mRNA stability and the expression of several genes including *Gfpt1*, is also involved in promoting GFAT1 Ser243 phosphorylation [35]. Since AUF1 is positively modulated by signals that enhance mTORC2 activation, these findings underscore the role of mTORC2 in modulating GFAT1 expression and Ser243 phosphorylation. How the presence of this phosphorylation could promote GFAT1 expression and/or stability remains to be further examined.

**Figure 3:**
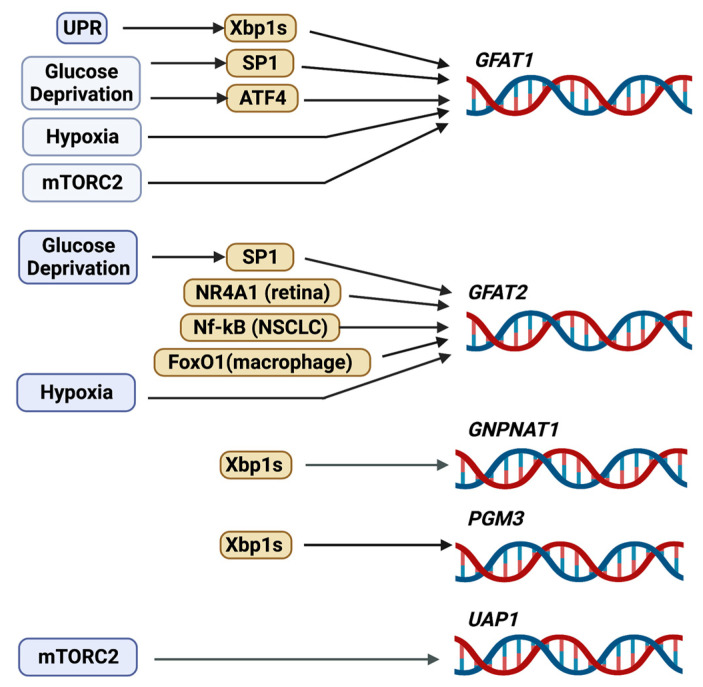
Transcriptional regulation of metabolic enzymes involved in the HBP.

Early studies have shown that GFAT1 gene expression is regulated by transcription factors such as Sp1 [36]. Although Sp1 binds to three elements of the *Gfpt1* proximal promoter (Figure 3), it is not clear how it mediates extracellular signals to induce the transcription of this gene. However, since Sp1 can bind to glucose response elements [37], it is conceivable that Sp1-mediated expression of *Gfpt1* occurs in response to nutrients such as glucose. As Sp1 also undergoes *O*-GlcNAcylation to inhibit its transcriptional activity [38], there is likely reciprocal regulation between Sp1 and de novo hexosamine biosynthesis. More recent studies support the notion that nutrient levels modulate GFAT1 expression. Glucose deprivation or the inhibition of glycolysis by using the glucose analog 2-deoxyglucose (2-DG) enhances both mRNA and protein expression of GFAT1. These stressful starvation conditions trigger the unfolded protein response (UPR), leading to increased activity of the transcription factor, spliced X-box-binding protein 1 (Xbp1s), which is translocated to the nucleus to augment the expression of *Gfpt1* [17,39,40]. Notably, pharmacological induction of the UPR also increases GFAT1 expression [17]. Another transcription factor that responds to nutrient stress, ATF4, is also required for increasing GFAT1 expression during glucose deprivation [17]. Indeed, the GCN2/eIF2α pathway triggers the expression and activation of ATF4 during nutrient limitation, such as glucose or leucine deprivation, which correlates with increased GFAT1 and cellular *O*-GlcNAcylation. In macrophages, GFAT1 mRNA is induced by hypoxia [41]. The GFAT promoter contains the consensus motif for the hypoxia response element HRE, but whether HIF1 binds to and controls GFAT1 transcription remains to be examined. The levels of GFAT1 mRNA are also decreased in the absence of SIN1, a component of mTORC2, indicating that it is modulated by this protein complex [40]. Since highly proliferating cells such as cancer cells have increased demand for nutrients to fuel their propagation, it is not surprising that GFAT1 expression is often upregulated in tumors (see Section 5.1). Nonetheless, while the regulation of GFAT1 expression and activation are fairly well studied, the mechanisms modulating its degradation are less well known. A half-life of 1h suggests a high rate of GFAT1 protein turnover [8]. How the latter is modulated would need to be investigated. 

GFAT also associates with signaling molecules that could modulate its activity or function. For example, recent studies reveal that GFAT1 interacts with mTORC2 and the tumor suppressor phosphatase known as PTEN, which triggers distinct cellular outcomes. On one hand, the upregulation and association of GFAT1 with PTEN in cervical cancer promotes the ubiquitination and degradation of the phosphatase while increasing cell proliferation [42]. Precisely how GFAT1 mediates PTEN degradation remains to be investigated. On the other hand, the presence of glutamine in the cell culture media controls the interaction of GFAT1 with mTORC2 in high-speed membrane-containing fractions [40]. Furthermore, GFAT1 associates with the transforming growth factor-β-activated kinase-1-binding protein 1 (TAB1) during glucose starvation [43]. The GFAT1-TAB1 interaction allows the formation of a complex with TTLL5, which is involved in the ligation of glutamate to substrates, such as TAB1. Indeed, the proximity of GFAT1 to TTLL5 in the newly formed GFAT1-TAB1-TTLL5 complex allows TTLL5 to use glutamate, a product of GFAT1 activity, to promote TAB1 glutamylation. The latter consequently enhances p38 MAPK activation and autophagy during glucose deprivation, thus increasing the survival of lung adenocarcinoma cell lines. The central role of GFAT1 in this process is underlined by its enzymatically inactive mutant, GFAT1 H577A, which is unable to rescue the growth of GFAT1-deleted cells during glucose starvation [43]. These findings suggest that GFAT1 may possess functions other than its canonical role in promoting de novo hexosamine production.

Compared to GFAT1, GFPT2/GFAT2 is relatively less studied. So far, the expression of GFAT2 has been reported in the CNS, in mouse embryonic stem cells, in cardiomyocytes, in macrophages, and in retinal and neuronal cells [44,45,46,47,48]. It is upregulated in several cancers including breast, ovarian, colon, leiomyosarcoma, and non-small cell lung cancer [49,50,51,52,53], and it is also enhanced in various tumors that are resistant to phagocytosis [54]. GFAT2 is slower than GFAT1 in synthesizing GlcN-6-P, but it is less sensitive to the feedback inhibition of UDP-GlcNAc [44,55,56]. The activity of GFAT2 is also increased by PKA-mediated phosphorylation of Ser202 (homologous to Ser205 of GFAT1) [27]. Similar to hGFAT1, hGFAT2 coalesces as tetramers and can also exist as higher-order oligomeric structures [55,57]. Whether GFAT1 and GFAT2 can form hetero-oligomers is not clear. Like GFAT1, Sp1 modulates the basal promoter activity of GFAT2 [58]. GFAT2 transcription is also modulated by distinct transcription factors. In the retina, the nuclear receptor subfamily 4 group A member 1 (NR4A1) modulates GFAT2 mRNA expression [47]. NF-kB transcriptionally upregulates GFAT2 in non-small cell lung cancer (NSCLC) [53]. In macrophages, FoxO1 mediates the effect of lipopolysaccharides (LPS) in inducing GFAT2 expression [46]. Furthermore, the amount of GFAT2 in pancreatic cancer has been linked to the expression of Stomatin-like protein 2 (SLP-2), a protein that is enhanced in this type of tumor and is associated with poor prognosis [59]. 

### 2.2. GNPNAT1/GNA

The enzyme GNPNAT1/GNA1 (glucosamine-phosphate *N*-acetyltransferase 1) uses acetyl-CoA to convert GlcN-6-P into *N*-acetylglucosamine-6-phosphate (GlcNAc-6-P) in the second step reaction of the HBP [60]. Gene inactivation of the murine *GNPNAT1* (*EMeg32*) is lethal for developing embryos at approximately day E7.5, while *EMeg32*-deficient embryonic stem cells (mESC) have decreased proliferation [61]. The surviving *EMeg32*-deficient murine embryonic fibroblasts have increased Akt activity and are more resistant to apoptotic stimuli. The transcription factor Xbp1s mediates the expression of *GNPNAT1* [39]. GNPNAT1 localizes to the cytoplasmic membrane leaflet of the Golgi and other intracellular membranes, and its protein levels increase during yeast mitosis. GNPNAT1 also associates with p97/valosin-containing proteins (VCP), which function in endocytosis. The increased expression and/or DNA methylation of *GNPNAT1* could serve as biomarkers for lung cancer and diabetes [62,63,64].

### 2.3. GNPDA

Similar to GNPNAT1/GNA1, the enzyme glucosamine-6-P deaminase (GNPDA1 and GNPDA2) uses GlcN-6-P as a substrate. However, instead of acetylating it to GlcNAc-6-P, they catalyze the deamination of GlcN-6-P to Fructose-6-P and ammonia, thus opposing the action of GFAT in providing intermediates for the HBP [65]. The metabolic consequences of counteracting the activity of GFAT1 via GNPDA1/2 are poorly understood. However, under particular conditions, GNPDAs can also reverse their direction of catalysis, which allows for the maintenance of UDP-GlcNAc levels [66]. GNPDAs are highly expressed in the hypothalamus and the adipose tissues [67,68]. Interestingly, in keratinocytes wherein GFAT1 is more predominantly expressed, knockdown of GNPDAs enhances the mRNA expression of GFAT2 [66]. In contrast, abrogating GNPDA2 in human adipose-derived mesenchymal stem cells alters the transcription of genes involved in lipid and glucose metabolism, suggesting that it also has a role in adipogenesis [69]. The expression of GNPDA2 but not GFAT decreases in the hypothalamus during high-fat diet [68]. In addition, while GNPDA2 is more expressed in chow-fed compared to fasted mice [70], its inhibition does not control appetite but instead, regulates glucose homeostasis. Thus, GNPDA2 could have a role in the CNS of modulating peripheral glucose levels. 

### 2.4. PGM3

Phosphoglucomutase 3 (PGM3) catalyzes the conversion of GlcNAc-6-P to GlcNAc-1-P in the third-step reaction of the HBP. Structural studies indicate that PGM3 has four functional domains that include a catalytic, a magnesium-binding, a sugar-binding, and a phosphate-binding domain. The catalytic domain undergoes phosphorylation at a serine in the third position of the sequence Ser/Th-X-Ser-His-Asn-Pro, which is conserved among phosphogluco-, phosphoglucosamine-, and phosphoacetylglucosamine mutases, whereas the Ser/Thr in the first position contributes to substrate specificity [71]. Phosphorylation of a second serine residue (Ser64) in the catalytic domain of PGM3 activates the enzyme in *E. coli* [72]. The *PGM3* gene is highly polymorphic in human populations and has served as a forensic and genetic marker since its early discovery [73]. Mutations in *PGM3* are found in combined immunodeficiency disorders, which underscores the importance of the HBP for a functional immune system (see Section 5.6). Similar to *GNPNAT1*, Xbp1s also mediates the transcription of *PGM3* [39]. PGM3 is essential for early embryonic development in mice, as its absence leads to defective implantation of the fetus [74]. Hypomorphic alleles of PGM3 that encode an enzyme with decreased activity result in reduced levels of UDP-GlcNAc in vivo. These mice have changes in pancreatic tissue architecture and are anemic, leukopenic, and thrombocytopenic. They also have defective spermatogenesis and glomerulonephritis. Aberrant glycosylation occurs for specific proteins such as a testis-specific isoform of angiotensin-converting enzyme (ACE), an integral membrane protein that normally undergoes heavy *N*- and *O*-glycosylation [74]. How the defective glycosylation of ACE could contribute to impaired spermatogenesis in the PGM3 hypomorphic mutant mice remains to be further explored. 

### 2.5. AMDHD2

The *N*-acetylglucosamine deacetylase (AMDHD2 or amidohydrolase domain containing 2) triggers the deacetylation of GlcNAc-6P to GlcN-6P, which is the reverse reaction catalyzed by GNPNAT1/GNA1 [75]. AMDHD2 consists of a deacetylase domain and a small domain with unknown function (DUF) that is important for its expression [44]. Indeed, mutations in the DUF domain diminish expression levels of the enzyme. Structural studies reveal that AMDHD2 is an obligate dimer. Whereas residues from both monomers are required for ligand binding in the active site, catalytic residues originate from one monomer only. Interestingly, a chemical mutagenesis of AMDH2 in mESC that induces the loss of its deacetylase activity results in the activation of the HBP [44]. GFAT2 rather than GFAT1 is expressed predominantly in these cells. Since GFAT2 is less sensitive to the negative feedback regulation by UDP-GlcNAc, a loss of AMDHD2 or its activity would consequently upregulate the de novo HBP and UDP-GlcNAc levels in mESCs. Whether AMDHD2 could have a more specific role in downregulating the HBP in other GFAT2-expressing cells deserves further investigation. In contrast, abrogating AMDHD2 in C2C12 or N2a cells, wherein GFAT1 is predominantly expressed, does not significantly increase UDP-GlcNAc levels [44], which underscores regulatory differences in the HBP controlled by either of the GFAT isoforms. 

### 2.6. UAP1

The last step in the HBP that reversibly converts UTP and GlcNAc-1P to UDP-GlcNAc and pyrophosphate is catalyzed by UDP-*N*-acetylhexosamine pyrophosphorylase 1 (UAP) (also called GlcNAc1P uridyltransferase) [76]. The human UAPs have two isoforms, AGX1 and AGX2, which result from alternative splicing of a single gene. AGX2 is distinguished by the presence of an extra 17 amino acid peptide [77]. Whereas AGX1 is 2-3-fold more active with GalNAc-1-P as a substrate, AGX2 is more active toward GlcNAc-1-P [78]. Structural studies support the distinct catalytic properties of these two isoforms [79]. Unlike GFAT1, UAP1 protein expression is not upregulated following glucose deficiency [17]. Its mRNA expression is decreased in SIN1-disrupted cells, suggesting it is modulated by mTORC2 [40]. UAP1 binds to the F-box protein, Fbxl17, and this interaction inhibits UAP1 phosphorylation and likely its activity [80]. In support of the latter, abrogating Fbxl17 enhances total *O*-GlcNAcylation of total proteins in breast cancer cells, implying increased UDP-GlcNAc generation. How the UAP1 gene and protein expression is regulated remains to be further examined. 

## 3. Salvage Nutrients 

In addition to de novo synthesis, UDP-GlcNAc can be generated by salvage mechanisms. Glucosamine (GlcN or GAM) and *N*-acetylglucosamine (GlcNAc or NAG) can be acquired from environmental sources or salvaged into the HBP via the degradation of glycoconjugates. The regulation of salvage mechanisms remains enigmatic. It is also unclear whether specific cell types preferentially utilize de novo versus salvage synthesis of UDP-GlcNAc. Since the salvage nutrients are used as dietary supplements and could have benefit for the treatment of disorders related to glycosylation, the regulation of the HBP salvage pathways and how they reprogram metabolism warrant further investigation.

### 3.1. Glucosamine

Glucosamine (GlcN) enters the HBP after hexokinases phosphorylate it on the carbon in the 6th position of the glucose ring, producing GlcN-6-P. Early cellular studies have shown that the majority of GlcN supplemented in cell culture media enters the HBP [81]. In isotope-tracing experiments, the addition of GlcN in human-colon-carcinoma cells increases the synthesis of UDP-GlcNAc and decreases the levels of UTP, suggesting increased flux through the HBP [82]. However, it remains unclear how the de novo hexosamine biosynthesis or GFAT1 activity could be affected by increased GlcN levels. Dietary GlcN supplementation of mice rescues some of the defects associated with the absence of GFAT1 in developing T cells [83]. Although it is not sufficient to fully restore the developmental block during the most highly proliferative stages of thymocyte development [double negative 3 (DN3)-DN4 and CD8-immature single-positive (CD8-ISP) stages], GlcN boosts the survival of DN and single-positive (SP) cells. However, only a slight increase in UDP-GlcNAc occurred in total thymocytes following this supplementation. Hence, whether the beneficial effects of GlcN supplementation in developing thymocytes are due to increased flux through the HBP or other indirect effects in metabolism remains to be investigated. 

Several lines of evidence support that GlcN impairs glucose metabolism. First, GlcN enters the cells via glucose transporters including GLUT1, GLUT2, and GLUT4. Whereas GLUT1 and GLUT4 display similar affinities for glucose and GlcN, GLUT2 has a higher affinity for GlcN than glucose [84]. Thus, GlcN and glucose compete for intracellular transport. Second, extended exposure to GlcN elevates the amounts of GlcN-6-P in adipocytes. In return, hexokinase is allosterically feedback-inhibited by the latter metabolite and, consequently, diminishes intracellular glucose transport [85,86]. Third, GlcN also reduces ATP levels in hepatoma cells [87]. Fourth, GlcN impairs mTORC1/Akt signaling. This is illustrated in retinal Müller cells wherein in vitro GlcN supplementation increases the phosphorylation of ER stress markers including PERK, eIF2α, and the expression of ATF4, which subsequently inhibits mTORC1 signaling via the upregulation of REDD1 [88]. Fifth, short-term administration of GlcN in vivo or in vitro cultured cells confers a phenotype with features of diabetes mellitus [8,89,90,91,92]. Together, these findings support a role for GlcN in dampening glucose metabolism and instigating insulin resistance. 

Despite the controversial effects of dietary GlcN, its health benefits need further inquiry. Since GlcN is highly concentrated in connective tissues and cartilage, it is widely used as a dietary supplement to prevent osteoarthritis and other inflammatory conditions [93]. The molecular mechanisms underlying its role in these specific diseases remain to be elucidated. It is noteworthy that GlcN also promotes lifespan in mice and lower organisms [87,94]. This latter effect of GlcN is due to its antagonism of glucose metabolism but is also independent of its role in the HBP. In this particular instance, GlcN instead promotes mitochondrial biogenesis and increases amino acid catabolism. Its anti-tumor effect also needs to be probed further [95,96,97]. Initial studies have shown that GlcN inhibits the growth of sarcoma and increases the survival of mice bearing this tumor, suggesting that this metabolite has chemotherapeutic properties [98]. In human prostate carcinoma cells, GlcN supplementation inhibits STAT3 signaling and prevents the expression of the STAT3 target survivin, an apoptosis inhibitor [99]. The inhibitory effect of GlcN on cell proliferation seems specific for cells in which STAT3 is constitutively active. In chronic myelogenous leukemia, GlcN promotes apoptosis via the translocation of cathepsin D and the downregulation of Bcl-xL [100]. More recent studies indicate that the use of GlcN decreases the risk of lung cancer in humans as well as its associated mortality [101]. Whether and how GlcN supplementation can reprogram the metabolism of highly proliferating (normal or tumor) cells by preventing glycolysis, which is usually upregulated during proliferation, and/or by promoting increased flux through the HBP would be important to address.

### 3.2. N-acetylglucosamine (GlcNAc)

The GlcNAc kinase or *N*-acetylglucosamine kinase (NAGK) phosphorylates its substrate *N*-acetylglucosamine (GlcNAc) to generate GlcNAc-6-phosphate [16,102,103]. NAGK, unlike GFAT1, is dispensable for embryonic mouse development [104]. However, it is involved in the regulation of Wnt signaling during the development of lower organisms and could, thus, potentially be involved in tissue-specific differentiation in higher eukaryotes [105]. NAGK is a member of the sugar-kinase/Hsp70/actin superfamily with a characteristic common ATPase domain. Nutrient-limited conditions enhance NAGK mRNA expression. In pancreatic ductal adenocarcinoma (PDAC), *NAGK* mRNA becomes upregulated during glutamine starvation [14]. *NAGK* mRNA is also increased in some cell lines under low glucose. However, despite the increase in mRNA, NAGK protein expression does not rise concomitantly during glutamine starvation. Nevertheless, NAGK may be modulated, presumably by phosphorylation, in response to glutamine levels [14]. The phosphorylation sites in NAGK remain to be identified. In platelets, it is phosphorylated at Tyr205, but the role of this phosphorylation is unknown [106]. How NAGK contributes to the HBP, particularly during normal growth conditions, is still unclear. When NAGK is deleted, de novo hexosamine biosynthesis increases [14], suggesting cross-talk between the salvage and de novo HBP. Consistent with this notion, glutamine deprivation suppresses de novo HBP but triggers NAGK-dependent salvage HBP in PDAC. Furthermore, NAGK is upregulated in these tumors and is required for their optimal growth. In PDAC, extracellular matrix components such as hyaluronic acid, which is composed of GlcNAc and glucuronic acid disaccharide units, provide sources of GlcNAc in the tumor microenvironment [107]. Thus, NAGK plays a role in supporting PDAC tumor growth by catalyzing the production of GlcNAc-6-P, which is used to generate UDP-GlcNAc. GlcNAc, which serves as a sugar subunit of the peptidoglycans from the bacterial cell wall, also activates the inflammasome in macrophages and dendritic cells during inflammation [108]. This involves the inhibition of hexokinase due to its dissociation from the outer membrane of the mitochondria. Inhibiting hexokinase could slow down glycolysis. Whether the increased GlcNAc could also affect NAGK and the HBP under these conditions is unclear. GlcNAc also affects other immune cells. For example, supplementation of mouse or human T cells in vitro with GlcNAc increases *N*-glycan branching [109]. However, GlcNAc salvage from the environment is inefficient due to a lack of specific cell surface transporter for this metabolite, and thus, its entry relies on macropinocytosis. In vitro acetylation of GlcNAc to generate GlcNAc-6-acetate can enhance its cell permeability and, thus, increase the synthesis of UDP-GlcNAc. This process favors the differentiation of activated T cells to anti-inflammatory T regulatory cells (Treg) over the generation of pro-inflammatory T helper (Th) Th1 and Th17 cells [109]. These findings suggest that enhancing the salvage pathway by increasing GlcNAc availability could be promising for the treatment of autoimmune diseases.

GlcNAc supplementation in mice augments their hepatic UDP-GlcNAc, as well as the *N*-glycan branching of hepatic glycoproteins [103]. It also increases body weight without affecting calorie intake and energy expenditure [103,110]. GlcNAc-treated mice may use equivalent calories more efficiently through increased nutrient absorption. Although supplementation with GlcNAc does not extend mice lifespan, there is improved memory in young male animals [110]. This effect is unlikely via the HBP since UDP-GlcNAc levels were not increased. Furthermore, the augmented expression of GFAT does not recapitulate this phenotype. Future studies should reveal how salvage metabolites such as GlcN and GlcNAc play essential roles in the central nervous system (CNS). As discussed further below (see Section 5.6), there are many neurological impairments that are characterized by defective glycosylation. Recently, it was discovered that brain glycogen serves as a reservoir for GlcN, thus further underscoring the importance of glycosylation in the CNS [111].

## 4. Functions

UDP-*N*-acetylglucosamine (UDP-GlcNAc) is the activated form of GlcNAc and serves as a substrate for *N*- and *O*-linked glycosylation. *N*-linked glycosylation of membrane and secretory proteins is critical for protein homeostasis (proteostasis) by mediating proper protein folding, stability, and function. *O*-GlcNAcylation of intracellular proteins modifies protein function, and these proteins act as signaling modulators. UDP-GlcNAc also plays major roles in the structural integrity of cells and tissues. It is used for the synthesis of chitin, peptidoglygans, and glycosaminoglycans [112,113]. It is also used for glycosylphosphatidylinositol (GPI) linkers that anchor cell surface proteins to the plasma membrane [114]. Mucin-type *O*-glycosylation of glycoproteins including membrane and extracellular matrix proteins also relies on UDP-GalNAc (epimer of UDP-GlcNAc) [115]. Some of these mucin-type *O*-linked glycoproteins can act as antigens to the immune system and, thus, have importance for the development of vaccines [116]. Here, we will discuss the functions of UDP-GlcNAc in the *N*-glycosylation of secretory and membrane proteins and how these functions are crucial for proteostasis. We will also discuss how UDP-GlcNAc is utilized for the *O*-GlcNAcylation of cytoplasmic and nuclear proteins and cite examples of key signaling molecules and metabolic enzymes that undergo *O*-GlcNAcylation.

### 4.1. Protein Homeostasis

**Figure 4:**
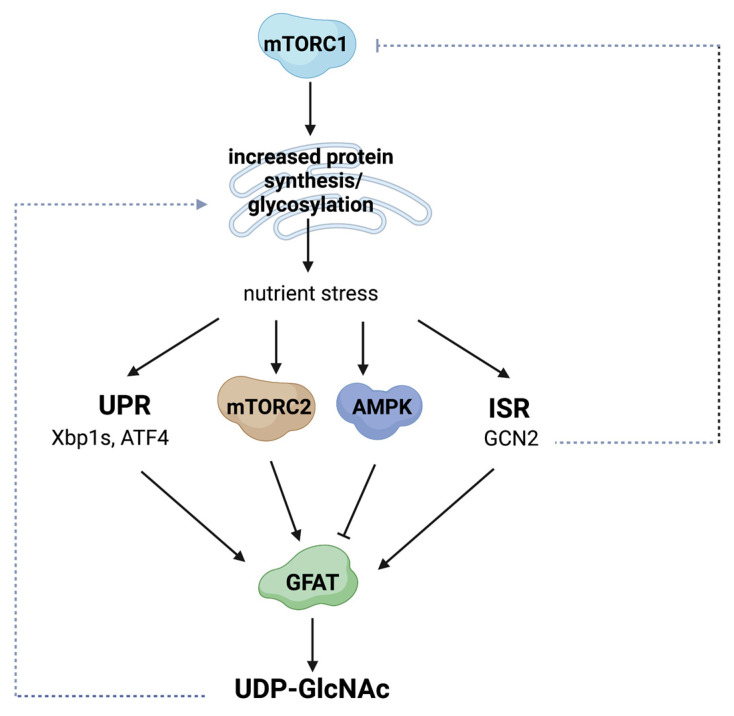
Conditions that increase demand for UDP-GlcNAc upregulate de novo HBP to maintain protein homeostasis.

In the ER, newly synthesized membrane and secretory proteins undergo glycosylation via the addition of glycans on Asn (*N*) residues. ER membrane-bound oligosaccharyltransferases co- or post-translationally transfer *N*-glycans with the core structure Glc_3_Man_9_GlcNAc_2_ (Glc—glucose; Man—mannose; GlcNAc—*N*-acetylglucosamine) from dolichol (non-sterol isoprenoids derived from the mevalonate pathway) to the Asn residues in N-X-S/T (X is not a Pro) motif of nascent polypeptides harbored in the ER lumen [117,118]. Glycan trimming and binding to the ER lectin chaperones calnexin or calreticulin facilitate protein folding in the ER. Further remodeling in the Golgi generates glycoproteins with complex *N*-glycans that can bind galectins. At the cell surface, the galectin–glycoprotein interactions form a macromolecular lattice that controls the clustering, signaling, and endocytosis/turnover of these glycoproteins. Several conditions that increase the demand for nutrients, such as elevated protein synthesis and glycosylation and/or profuse mTORC1 signaling (e.g., oncogenic mutations that increase mTORC1 signals) trigger ER stress responses including the unfolded protein response (UPR) (Figure 4). Three ER transmembrane proteins, namely, activating transcription factor 6 (ATF6), inositol-requiring enzyme 1 (IRE1), and double-stranded RNA-activated protein kinase (PKR)-like ER kinase (PERK), respond to the induction of ER stress [119]. The activation of each of these protein misfolding stress sensors upregulates the transcription factors ATF6, Xbp1s, and ATF4, respectively. These transcription factors induce the expression of ER chaperones and quality control factors that alleviate protein misfolding. The ER stress response also diminishes mRNA translation and enhances ER-associated protein degradation (ERAD) to restore proteostasis and, consequently, cellular homeostasis.

Increased flux through the de novo HBP requires upregulation of GFAT1 to maintain proteostasis during nutrient stress conditions [40,120]. For example, both GFAT1 and Xbp1s are necessary for cardio-protection following ischemia reperfusion in the heart. During the latter, UPR is triggered, along with the activation of the HBP and increased *O*-GlcNAc protein modification [121]. Xbp1s induces *Gfpt1* transcription, while the HBP reciprocally modulates the UPR. ATF6 undergoes *N*-linked glycosylation at three evolutionarily conserved sites [122]. ATF6 *N*-glycosylation senses ER homeostasis and triggers the UPR, highlighting the role of de novo HBP during stress response. Furthermore, the integrated stress response (ISR), which activates GCN2 (general control nonderepressible 2) during amino acid deprivation also regulates GFAT1. The activation of GCN2 also negatively modulates mTORC1 signaling to dampen protein synthesis [123]. In addition, nutrient limitation also activates mTORC2, which modulates the phosphorylation and protein stability of GFAT1 [12]. By modulating GFAT1, mTORC2 coordinates nutrient fluctuations with the activation of the de novo HBP. In contrast, AMPK-mediated phosphorylation of GFAT1 diminishes its activity [32,33]. It is not clear how this AMPK function could mitigate ER stress, but it could instead have indirect metabolic consequences that would subsequently diminish nutrient demand. The role of de novo HBP in maintaining proteostasis is further highlighted by recent studies that abrogate GFAT1 in vivo. In mice harboring specific deletion of GFAT1 in thymocytes, the UPR and ISR increase [83]. In these cells, the deficiency of GFAT1 enhances the proportion of oligomannose-type *N*-glycans, diminishes total *O*-GlcNAcylation, and impairs T cell receptor glycosylation and expression. GlcN partially restores some of the developmental defects associated with the loss of GFAT1 but only scantly rescues UDP-GlcNAc levels. These findings underscore the need for the de novo HBP in maintaining proteostasis and proper protein glycosylation when demands for metabolites are augmented during cell proliferation and development.

The function of the HBP in maintaining proteostasis may be relevant in aging. Increased activation of the HBP reduces proteotoxicity and increases lifespan in *C. elegans* [124,125]. The expression of gain-of-function mutations of GFAT1 in cultured mouse neuronal cells or their supplementation with GlcNAc diminishes the aggregation of the polyglutamine (polyQ) protein Ataxin-3. Increasing HBP activity in these cells also promotes the ISR via increased phosphorylation of PERK and eIF2a as well as the activation of ATF4. How the augmented ISR could improve proteostasis remains to be further examined, but it could involve increased protein turnover or decreased protein synthesis and/or reduced formation of toxic protein aggregates [125].

### 4.2. Protein Modification by O-GlcNAcylation

*O*-GlcNAcylation (*O*-linked β-*N*-acetylglucosamine) of cytoplasmic and nuclear proteins has numerous roles in biological processes including cell proliferation, activation of the immune system, apoptosis, stress response, protein trafficking, and nutrient sensing. The product of the HBP, UDP-GlcNAc, serves as a substrate for protein *O*-GlcNAcylation. There have been many recent excellent reviews discussing *O*-GlcNAcylation [126,127,128,129,130]. What remains controversial is how nutrient availability controls the HBP and *O*-GlcNAcylation. Here, we focus our discussion on this issue and cite examples of metabolic and signaling targets of *O*-GlcNAcylation in response to nutrients.

**Figure 5:**
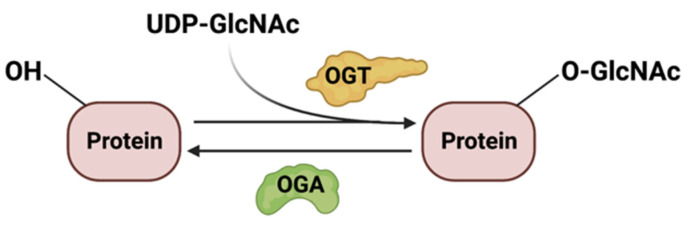
The *O*-GlcNAcylation of proteins is dynamically regulated by *O*-GlcNAc transferase (OGT) and *O*-GlcNAcase (OGA).

*O*-GlcNAcylation is dynamically regulated by *O*-GlcNAc transferase (OGT) and *O*-GlcNAcase (OGA) (Figure 5), which adds and removes glycosylation at the Ser/Thr sites of glycoproteins, respectively. Since *O*-GlcNAcylation relies on the availability of UDP-GlcNAc, this modification is, thus, dependent on flux through the HBP. However, this is not the only factor that modulates protein *O*-GlcNAcylation. Indeed, the availability of nutrients, the activity and abundance of OGT and OGA enzymes, and the amounts of substrates also regulate this process. 

Early studies reveal that increased flux through the HBP or increasing the concentration of UDP-GlcNAc augments *O*-GlcNAcylation of various substrates [131]. Since the HBP is dependent on the availability of glucose, glutamine, acetyl-CoA, and UTP, increased *O*-GlcNAcylation could occur when these nutrients are elevated [132]. In highly proliferating cells such as cancer cells, exposure to high glucose upregulates the HBP and increases *O*-GlcNAcylation, which is linked to tumor aggressiveness and/or metastasis [133]. However, many studies have also shown that glucose deprivation or the inhibition of glucose metabolism enhances cellular protein *O*-GlcNAcylation [12,17,134,135,136]. Consistent with these findings, the activation of AMPK, which occurs during energy depletion, also increases cellular *O*-GlcNAcylation [134]. Hence, total protein *O*-GlcNAcylation is responsive to high or low glucose levels, which are nutrient stress conditions that trigger HBP activation. The levels of glutamine also affect the HBP and, thus, the levels of UDP-GlcNAc and *O*-GlcNAcylation. *O*-GlcNAcylation of total proteins is downregulated during glucose deprivation when glutamine is limited [12], supporting the requirement for glutamine in the HBP. Furthermore, diminished glutamine levels favor glycolysis over glutaminolysis in the white adipose tissue of obese individuals. This metabolic reprogramming increases UDP-GlcNAc levels, as well as the specific *O*-GlcNAcylation of chromatin-binding proteins that activate inflammatory genes, while total cytosolic proteins are not affected [137]. In contrast, conditions that trigger the ISR such as amino acid shortage and GCN2 activation increase the expression of ATF4, which modulates the amount of GFAT1. Such conditions, including the withdrawal of leucine, enhance protein *O*-GlcNAcylation [17]. Clearly, more work is needed to delineate how nutrient levels link flux through the HBP with the *O*-GlcNAcylation of total proteins. Future studies should elucidate how metabolic reprogramming due to fluctuating levels of nutrients that have input into the HBP (e.g., acetyl-CoA and UTP) impact the *O*-GlcNAcylation of total and specific proteins. 

The HBP also modulates other metabolic pathways via *O*-GlcNAcylation of key regulatory molecules. Several glycolytic enzymes undergo *O*-GlcNAcylation to influence the flux through glycolysis as well as impinge on metabolic pathways [138]. For example, the *O*-GlcNAcylation of phosphofructokinase 1 (PFK1) inhibits its activity and reroutes the flux of glucose through the pentose phosphate pathway instead of glycolysis [138]. In contrast, *O*-GlcNAcylation of PKM2, a splice isoform of pyruvate kinase, is associated with increased glucose consumption and enhanced aerobic glycolysis (Warburg effect) [139]. *O*-GlcNAcylation of SRPK2 (serine-/arginine-rich protein kinase 2) regulates de novo lipogenesis by controlling the pre-mRNA splicing of lipogenic genes [140]. SRPK2 *O*-GlcNAcylation occurs at a nuclear localization signal motif that allows the import of this protein into the nucleus. Its *O*-GlcNAcylation is dependent on GFAT1 and increased levels of UDP-GlcNAc. The GFAT1/HBP-mediated *O*-GlcNAcylation of the ribonucleotide reductase (RNR) impairs its activity and leads to defective nucleotide metabolism [141]. Notably, the decreased dNTP pools under these conditions can induce KRAS mutations in pancreatic cancer cells. While leucine withdrawal enhances total protein *O*-GlcNAcylation [17], glucose starvation induces *O*-GlcNAcylation of the intracellular leucine sensor leucyl-tRNA synthetase 1 (LASRS1) on Ser1042 [142]. This modification diminishes the affinity of LARS1 for leucine while preventing its interaction with the RagD GTPase and decreasing mTORC1 activity. Hence, the glycosylation of LARS1 integrates the availability of glucose and leucine to regulate mTORC1. Together, these findings highlight how *O*-GlcNAcylation serves to link the HBP with the availability of nutrients and the coordination of other metabolic pathways or processes.

Signaling molecules that respond to nutrients and that have been linked to HBP regulation are also modulated via *O*-GlcNAcylation. Some of the *O*-GlcNAcylated Ser/Thr residues have been found to undergo phosphorylation as well, while others are localized close to phosphosites. Hence, the dynamic regulation of these proteins via *O*-GlcNAcylation versus phosphorylation occurs in response to environmental and cellular conditions. For example, c-Myc, an oncogenic transcription factor, is *O*-GlcNAcylated at Thr58, a residue that is also phosphorylated and frequently mutated in lymphoma [143]. Serum starvation enhances Thr58 glycosylation and the protein stability of c-Myc while decreasing its phosphorylation [144]. In contrast, the inhibition of OGT or conditions that affect flux through the HBP such as glucose depletion diminish the expression of c-Myc protein but not mRNA, indicating that *O*-GlcNAcylation modulates c-Myc post-transcriptionally [145,146]. Another transcription factor, FoxO1, which is negatively modulated by Akt, is *O*-GlcNAcylated in response to glucose [147]. This modification in FoxO1 increases expression of gluconeogenic genes as well as genes involved in detoxifying reacting oxygen species and could thus have a metabolic as well as protective function. Moreover, the protein kinase Akt, the activity of which is often upregulated in cancer, undergoes *O*-GlcNAcylation at Thr305 and Thr312 [148]. Glycosylation at these sites prevents the phosphorylation of Akt at Thr308, a site that is required for its activation by PDK1. It is also *O*-GlcNAcylated at Thr430 and Thr479, and these modifications enhance AktSer473 phosphorylation and the activity of this kinase [149]. Reciprocally, *O*-GlcNAcylation is modulated via the mTOR/Akt/c-Myc pathway via regulation of the expression of OGT [150]. On one hand, the activation of Akt and mTOR elevates OGT and *O*-GlcNAcylation in breast cancer cells. On the other hand, the regulation of OGT by *c*-Myc requires HSP90A, a transcriptional target of *c*-Myc. Lastly, epigenetic modulation, which includes DNA and histone modifications that alter chromatin structure and gene expression, as well as translation regulation, are wired to the HBP through *O*-GlcNAcylation of the key signaling molecules in these processes [151]. Future studies should elucidate how nutrient availability could control the expression of a specific set of genes via *O*-GlcNAcylation of these signaling proteins, translation, and transcription/epigenetic modulators.

## 5. HBP in Health and Disease 

**Figure 6:**
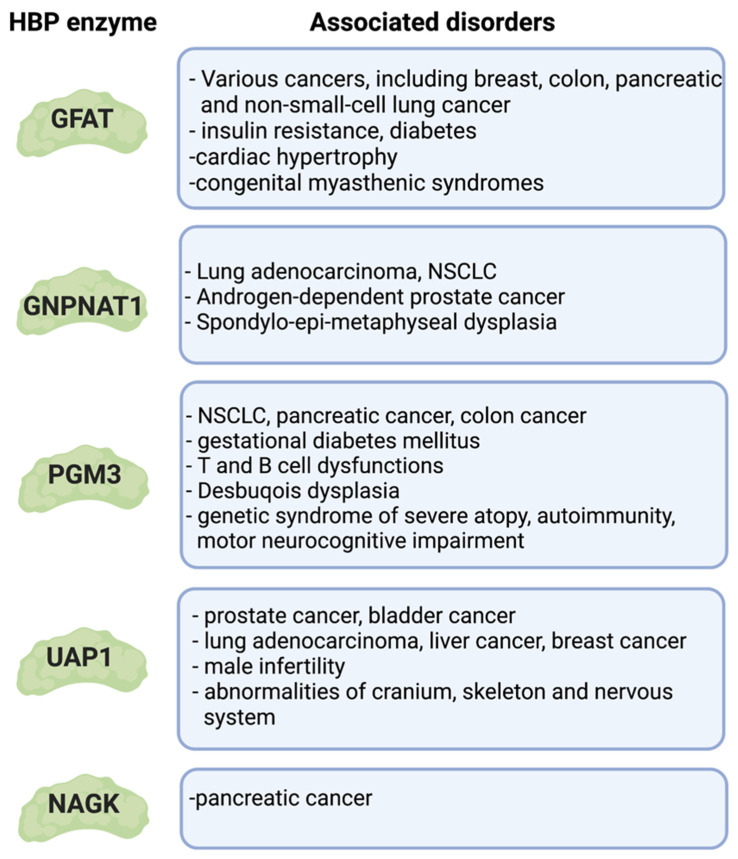
Alterations in the expression and/or regulation of the HBP enzymes have been linked to a variety of diseases.

Various pathological conditions that have underlying defects in the HBP underscore the importance of this pathway for the proper regulation of metabolic homeostasis and protein/lipid glycosylation (Figure 6). Oncogenic mutations in cancers reprogram their metabolism and often upregulate the HBP. Excess or limitation of nutrients also impacts the HBP and could trigger insulin resistance and diabetes. Specific cell types could also be more vulnerable to the improper glycosylation of proteins. For example, receptor *N*-glycan branching on the surface of immune cells relies on sufficient amounts of UDP-GlcNAc, such that defects in this process underlie a variety of immune-related disorders. Lastly, genetic mutations in key HBP enzymes occur in congenital disorders of glycosylation (CDG) in humans. Understanding how the HBP is deregulated in these conditions should provide insights on more effective therapeutic strategies to combat these diseases. 

### 5.1. Cancer

Many studies have assessed the levels of GFAT1/GFAT2 and other metabolic enzymes involved in the HBP in various cancer cells. The levels of these enzymes are often upregulated and correspond to increased HBP flux [49,152], enhanced levels of UDP-GlcNAc, and elevated *O*-GlcNAcylation of total proteins. To wit, the expression of GFAT1 and/or GFAT2 is augmented in breast, colon, pancreatic, and non-small-cell lung (NSCLC) cancer [153,154,155]. This increased expression is associated with specific oncogenic mutations or metabolic reprogramming. Upregulation of the HBP and GFAT2 occurs in mice and human NSCLC that expresses mutant KRAS/LKB1 [155]. Oncogenic KRAS also promotes the flux of glucose through the HBP in pancreatic ductal adenocarcinoma (PDAC) [156], while hypoxia increases the expression of GFAT1/GFAT2 in these cancer cells [157]. Upregulation of the HBP is also linked to the overexpression of hyaluronan (HA), a matrix protein that is involved in promoting tumorigenesis [158]. Increased flux through the HBP augments HA biosynthesis in breast cancer cells, resulting in enhanced HIF1α signaling, which further accelerates HBP flux and promotes cancer-stem-cell-like properties [158]. As a substrate for the salvage pathway, HA can also be used to generate UDP-GlcNAc and allow PDAC to grow when GFAT1 is abrogated in these cells [107]. Notably, PDAC in particular requires the salvage enzyme NAGK under glutamine-limited conditions, wherein de novo biosynthesis is downregulated [14]. Together, these findings provide support that increased flux through the HBP, via de novo or salvage synthesis, could play a role in cancer growth and progression. Indeed, inhibiting GFAT is a promising strategy for cancer therapy (see also Section 6). Pharmacological inhibition or gene silencing of GFAT1 or GFAT2 blocks the proliferation of cancer cells and reduces their invasive capacity, which is consistent with a role for GFAT in tumor growth and malignancy [152,155]. Targeted inhibition of GFAT in cancer cells may additionally affect the tumor microenvironment (TME). For instance, the inhibition of GFAT1 by glutamine analogs such as 6-diazo-5-oxonorleucine (DON) diminishes the levels of HA and collagen in the TME of PDAC [159]. This facilitates and increases the infiltration of CD8 T cells and sensitizes the pancreatic tumor to anti-PD1 immunotherapy. *O*-diazoacetyl-*L*-serine (azaserine) is another glutamine analog that inhibits GFAT1/2. GFAT2 is overexpressed in various phagocytosis-resistant tumors. Notably, treating colon cancer cells that overexpress GFAT2 with azaserine improves the efficacy of anti-CD47 therapy, consequently enhancing their phagocytosis by macrophages [54]. These findings highlight the metabolic plasticity of tumors and the role of the tumor microenvironment in supporting the nutrient/metabolic needs of the growing tumor. Hence, inhibiting the de novo and salvage hexosamine biosynthesis may be necessary to prevent tumor growth as well as enhance anti-tumor immune responses.

Besides GFAT, a number of other HBP enzymes also play a role in tumorigenesis or cancer growth. The expression of GNPNAT1 is upregulated in lung adenocarcinoma (LUAD) and correlates with DNA copy amplification, low DNA methylation, and poor prognosis [160]. Increased GNPNAT1 expression is also predictive for poor prognosis in NSCLC [161,162]. GNPNAT1 (along with UAP1) mRNA is also elevated in androgen-dependent prostate cancer and corresponds with increased levels of UDP-GlcNAc. These findings further support that upregulation of the HBP promotes cancer progression. In contrast, the HBP is downregulated in castration-resistant prostate cancer (CRPC) [163]. This downregulation is associated with increased PI3K/Akt signaling and SP1-ChREBP activity. Interestingly, supplementation of CRPC with UDP-GlcNAc decreases cell proliferation in vitro and in vivo. Precisely how the addition UDP-GlcNAc in CRPC could reprogram the metabolism that may explain the reduced cell proliferation would need to be interrogated.

PGM3 also plays a role in cancer. For example, there is enhanced dependence on PGM3 (as well as GFAT2) in NSCLC bearing mutations in KRAS and LKB1 [155,164]. The PGM3 competitive inhibitor known as FR054 attenuates EGFR-Akt signaling and triggers ER stress and ROS, leading to diminished tumor growth and increased cell death of KRAS/LKB-mutant NSCLC cells in vitro and in vivo. PGM3 is also overexpressed in human pancreatic cancer tissues, and its upregulation is associated with poorer median overall survival [165]. Gemcitabine-resistant pancreatic cancer has upregulated PGM3. Furthermore, PGM3 is upregulated in colorectal cancer tissues [166], where it maintains β-catenin activity by elevating protein *O*-GlcNAcylation. 

UAP1 is overexpressed in several cancers. Its upregulation protects prostate cancer cells from ER stress, resulting in a growth advantage [167]. UAP1, along with other genes involved in glycosylation, undergoes changes in expression during androgen stimulation or deprivation [168]. Cells with high UAP1 levels have a 10-fold increased amount of UDP-GlcNAc. They are also associated with resistance to inhibitors of *N*-linked glycosylation such as tunicamycin but not against a general ER stress-inducing agent, the calcium ionophore A23187 [167]. Depletion of UAP1 in these cells re-sensitized them to inhibitors of *N*-linked glycosylation, thus underscoring the role of UAP1 in generating UDP-GlcNAc that is critical for tumor growth. UAP1 is also upregulated in bladder cancer cell lines [169]. Silencing of UAP1 in these cells reduces their proliferation in vitro. Furthermore, UAP1 overexpression correlates with metastasis and poor prognosis of osteosarcoma patients [170]. Bioinformatics-based studies reveal that UAP1 is upregulated in lung adenocarcinoma, and its high expression correlates with a poor clinical outcome [171]. The analysis of signaling pathways that are enriched with genes related to the overexpression of UAP1 suggests that they play major roles in amino and nucleotide sugar metabolism, in the signaling of aminoacyl-tRNA biosynthesis, and in protein export. Similar to PGM3 in colorectal cancer [166], the upregulation of UAP1 in liver cancer correlates with increased *O*-GlcNAcylation and overexpression of β-catenin [172]. UAPL1, a paralog of UAP1 that shares 59% sequence identity, is also upregulated in tumors such as hepatocellular carcinoma (HCC) and prostate and breast cancer [173,174,175]. Although UAPL1 directly interacts with OGT and, like UAP1, is required for OGT-mediated protein *O*-GlcNAcylation, it has only a minor role in UDP-GlcNAc synthesis. Collectively, as the above findings reveal, UAP1 expression has a critical role in the HBP and tumor growth. Hence, inhibitors of this enzyme are being developed as anticancer agents [176,177].

Upregulation of the HBP could allow for the expression of essential cell surface proteins that promote growth and proliferation. In particular, the expression of growth factor receptors that critically regulate tumorigenesis could be enhanced [2]. For instance, the epidermal growth factor receptor (EGFR) is upregulated in several types of cancer. EGFR and other receptors that promote cell growth tend to have a high number of *N*-glycans, whereas cell surface proteins that are growth-inhibitory have low amounts of glycosylation [178]. *N*-glycan branching is highly sensitive to the levels of hexosamine, and thus, it is also contingent on increased HBP flux. The HBP, along with the coat complex II (COPII), becomes upregulated to promote the survival of lung adenocarcinoma (LUAD) but not lung squamous carcinoma (LUSC) cells during glucose limitation [179]. This sustains the expression of a subset of cell surface proteins, including EGFR, under these conditions. Indeed, augmented EGFR activation correlates with increased GFAT1 expression in LUAD patient samples. Thus, it would be worthwhile to identify and characterize tumor-specific cell surface proteins that are particularly dependent on increased HBP flux for their expression, as they could serve as useful biomarkers or therapeutic targets.

Many types of cancer display increased protein *O*-GlcNAcylation [180]. Elevated *O*-GlcNacylation is linked to metastatic breast, prostate, colon, lung, and other cancers [181,182,183,184]. This is usually accompanied by altered expression of the *O*-GlcNAc cycling enzymes referred to as OGT and OGA [185]. The expression of OGT promotes the growth of cancer stem cells (CSC) in colon cancer, whereas OGA inhibits this growth [186]. Increased *O*-GlcNAcylation is, therefore, associated with the upregulation of CSC subsets. Deregulation of protein *O*-GlcNAcylation can also promote tumor growth and metastasis. Several key proto-oncogenes undergo *O*-GlcNAcylation, which modulates their expression and/or activity (see Section 4.2) [187]. Metabolic enzymes also undergo *O*-GlcNAcylation, which can enhance tumorigenesis via metabolic reprogramming. For example, *O*-GlcNAcylation of the pyruvate kinase M2 (PKM2) under high glucose conditions inhibits its catalytic activity, promoting aerobic glycolysis and tumor growth [188]. Furthermore, proteins involved in the epithelial–mesenchymal transition (EMT) and the extracellular matrix (ECM) undergo deregulated *O*-GlcNAcylation to promote tumor cell motility, invasion, and metastasis [181,189]. OGT and OGA also interact with epigenetic regulators, histones, and histone-remodeling complexes and could thereby alter the expression of genes involved in tumorigenesis and cancer progression [190,191]. For instance, *O*-GlcNAcylation epigenetically regulates the expression of the proto-oncogene MYBL1, which reduces colon cancer stem cell and tumor growth. Modulating the cycling of OGT and OGA via pharmacological means could thus show promise in preventing the growth and metastasis of cancer cells [182,183]. 

### 5.2. Insulin Resistance and Diabetes

Initial studies have demonstrated that increased flux through the HBP impairs glucose transport, a hallmark of insulin resistance [192,193]. The finding that glutamine, a substrate (along with fructose-6-P) of de novo HBP, is required for the desensitization to insulin stimulation further strengthened the notion that the de novo HBP is involved in sensing glucose and the induction of insulin resistance [8]. Treatment with glutamine analogs that inhibit GFAT1 also prevents insulin resistance during high glucose exposure of adipocytes. Furthermore, GlcN infusions of healthy animals result in augmented UDP-GlcNAc concentrations in their muscles while impairing the utilization of glucose [194]. The levels of UDP-GlcNAc are also elevated in skeletal muscles during hyperglycemia and hyperinsulinemia [195,196]. Moreover, increased *O*-GlcNAcylation occurs in tissues of diabetic or insulin-resistant rats [197]. Hence, these findings hint that increased flux through the HBP and elevated UDP-GlcNAc production correlate with insulin resistance.

The upregulation of GFAT1 that occurs during insulin resistance and/or diabetes results in augmented flux through the HBP. Patients with type 2 diabetes have elevated GFAT1 activity in biopsies of skeletal muscle. Furthermore, increased GFAT activity in patients is associated with postprandial hyperglycemia, oxidative stress, and diabetic complications [198,199]. Increased expression of GFAT1 in fat and skeletal muscle of transgenic mice modestly enhances the levels of UDP-GlcNAc, but it is sufficient to promote insulin resistance [200,201]. Furthermore, transgenic mice that overexpress GFAT1 in the liver have increased glycogen storage and hyperlipidemia. They develop insulin resistance, ultimately leading to increased body weight and obesity [202]. Elevated GFAT1 levels in HepG2 liver cells also augment the expression of lipogenic genes [203]. The molecular mechanisms by which GFAT1 is upregulated during insulin resistance remain to be investigated, but a putative mediator is Xbp1s. This transcription factor is induced by ER stress, and it is deregulated in metabolic diseases [204]. Insulin resistance/diabetes is closely linked to ER stress, which is coupled to increased HBP [203,205]. Overnutrition deregulates glucose and lipid metabolism and, in turn, triggers chronic ER stress [206]. Transcriptional regulators such as Xbp1s, which is induced by nutrient stress, modulate both ER stress responses and de novo HBP [39,205]. Deficiency in Xbp1 in mice results in insulin resistance, further supporting that the Xbp1/ER stress/HBP axis plays a pivotal role in maintaining insulin sensitivity [205]. How the HBP is reprogrammed over time, leading to insulin resistance and diabetes, would need to be further examined. Whether altered expression or activity of other enzymes involved in the catalysis of the HBP occurs in or leads to insulin resistance/diabetes also needs to be investigated. So far, changes in the expression of PGM3 have been linked to gestational diabetes mellitus [207].

Despite accumulating evidence linking elevated HBP flux to insulin resistance/diabetes, the underlying mechanisms remain poorly understood. The increased flux of glucose through the HBP can blunt glucose transport, particularly in insulin-responsive adipose tissue and skeletal muscle [208]. Mice overexpressing GFAT1 in adipocytes and skeletal muscle develop insulin resistance due to defective GLUT4 translocation or docking into the cell membrane [200,209]. The treatment of skeletal muscle or adipocytes with the salvage metabolite, GlcN, also inhibits glucose transport by preventing the translocation of GLUT4 to their cell membrane [89,194,210]. These findings raise the question as to how the HBP affects glucose transporters. Aberrant *N*-glycosylation of pancreatic β-cell GLUT2 impairs insulin secretion in type 2 diabetes [211,212]. GLUT4 is *N*-glycosylated, and the absence of this modification prevents its subcellular localization during insulin stimulation [213]. Thus, elevated HBP flux could affect the *N*-glycan branching of glucose transporters. Munc18c, a syntaxin 4-binding protein that regulates the docking/fusion step of glucose transporters, also undergoes *O*-GlcNAcylation [214,215]. Hence, this modification could modulate the function of Munc18c. Notably, many studies have addressed the role of *O*-GlcNAcylation in insulin resistance and diabetes since this protein modification depends partly on the levels of UDP-GlcNAc. Mice lacking OGT in pancreatic β-cells develop diabetes and β-cell failure [216]. OGT is indispensable for mediating insulin secretion and cell survival. Reconstituting Akt signaling and alleviating ER stress in β-cells rescues the phenotype associated with the loss of OGT. Furthermore, abrogating OGA in pancreatic β-cells also leads to impaired glucose homeostasis in vivo [217]. Thus, balanced expression of OGT and OGA in β-cells is necessary for the proper levels of *O*-GlcNAcylation. Defective *O*-GlcNAcylation of proteins involved in insulin signaling has been associated with hyperglycemia, insulin resistance, and diabetes [218]. For example, IRS-1 *O*-GlcNAcylation diminishes its interaction with PI3K p85 and, thus, reduces Akt signaling [219]. PI3K effectors such as PDK1 also undergo *O*-GlcNAcylation, although the effect of *O*-GlcNAcylation on the activity of PDK1 will need to be further examined [219]. Akt is *O*-GlcNAcylated at several sites, including Ser473, a residue that is also phosphorylated by mTORC2 [220]. Intriguingly, the *O*-GlcNAcylation of Ser473 increases the kinase activity of Akt in vitro, but the function in vivo remains to be delineated. Transcriptional regulators of gluconeogenesis and lipogenesis, such as FoxO1, PGC1α, CRTC2, LXR, and ChREBP, become *O*-GlcNAcylated, and this modification is linked to high glucose-triggered expression of their target genes [218,221,222]. The transcriptional regulator p53 also undergoes *O*-GlcNAcylation, and this modification stabilizes p53 during starvation [223]. p53 binds the promoter of PCK1 to control hepatic gluconeogenesis. A number of metabolic enzymes are also subject to *O*-GlcNAcylation. Thus, glucokinase (GCK; hexokinase IV) becomes *O*-GlcNAcylated in the liver, and its level of expression in the *ob/ob* mice correlates with their amount of *O*-GlcNAc [224]. Despite a strong correlation of increased *O*-GlcNAcylation during insulin resistance/diabetes, some studies indicate that reducing this protein modification (by inhibiting OGT or overexpressing OGA) in vitro is not sufficient to prevent insulin resistance in adipocytes [225]. Nevertheless, the correlative relationship between the levels of protein *O*-GlcNAcylation and insulin resistance could be exploited for pre-diabetes and diabetes screening and/or diagnostic purposes [226]. 

### 5.3. Immunity

The HBP plays various roles in the innate and adaptive immune system. Glycosylation of cell-surface and intracellular proteins regulates the development and signaling of immune cells, as well as the recognition of pathogens and antigens. Defective glycosylation is found in various immune-related disorders. Understanding the role of the HBP and glycosylation in immunity has many implications for the development of more effective vaccines, the treatment for immune-related disorders, and anti-tumor immunity.

In humans, mutations of PGM3 occur in severe immunodeficiencies that are characterized by T and B cell dysfunctions [227,228,229,230]. PGM3 defects impair UDP-GlcNAc biosynthesis and *N*-glycan structures, and thus, the disease presents not only with a deregulated immune system but with defects in other organs as well [231]. Whether other enzymes of the HBP, such as UAP1, could be mutated in other immunodeficiency disorders and the precise underlying aberrant mechanisms remain to be elucidated.

Accumulating evidence from genetic studies that abrogate key HBP enzymes supports the role of this metabolic pathway in T cell development and function. During early T cell ontogeny in the thymus, de novo HBP is required for the proper development of αβ-T cells [83]. The abrogation of GFAT1 diminishes the levels of UDP-GlcNAc and impairs complex *N*-glycosylation of TCRβ on the surface of developing αβ thymocytes while increasing the proportion of oligomannose-type *N*-glycans. The loss of the de novo HBP also augments ER stress and the integrated stress response. Interestingly, γδ-TCR diversity is compromised, although γδ-T cells have increased cell numbers in the absence of GFAT1. Dietary supplementation of mice with GlcN and an α-ketoglutarate analog enhances the survival of double-negative (DN) and single-positive (SP; CD4^+^ or CD8^+^) cells but does not alleviate the developmental block during the highly proliferative DN3-DN4 and CD8-ISP stages. Cells of the latter stages are involved in TCRβ selection and TCRα recombination. Hence, in the absence of de novo HBP, salvage synthesis of UDP-GlcNAc could only rescue the survival of SP and DN cells, but it is insufficient to sustain normal αβ-T cell development. Studies that ablate Mgat1 (α-1,3-mannosyl-glycoprotein 2-β-*N*-acetylglucosaminyltransferase), a Golgi enzyme that initiates *N*-glycan branching, demonstrate that this glycan modification is required for positive selection, thus promoting central tolerance during T cell development. Indeed, *N*-glycan branching of the TCR and co-receptors facilitate their binding to low-affinity peptide-MHC while preventing high-affinity interactions [232]. Together, these findings emphasize the role of the HBP, which provides UDP-GlcNAc, which is crucial for *N*-glycosylation, during early T cell development.

The requirement for glycosylation during peripheral T cell differentiation and activation has been extensively studied by ablating enzymes involved in *N*-glycan branching, a process that is sensitive to UDP-GlcNAc levels. These studies reveal that *N*-glycan branching inhibits TCR clustering and signaling, thus negatively regulating T cell activation and autoimmunity [232,233]. In both mice and humans, decreased *N*-glycan branching promotes the growth of pro-inflammatory Th17 cells and autoimmunity while preventing anti-inflammatory Treg cell differentiation. Increasing aerobic glycolysis and glutaminolysis during T cell activation diminishes the flux through the HBP and, consequently, reduces UDP-GlcNAc levels and *N*-glycan branching [9]. Patients with ulcerative colitis (UC) have pro-inflammatory mucosal T lymphocytes, which is in line with the phenotype conferred by decreased *N*-glycan branching. Ex vivo supplementation of these cells with GlcNAc increases TCR *N*-glycan branching, represses T cell growth, and prevents Th1/Th7 immune responses [234]. Mice that have deficiency in *N*-glycan branching are also more susceptible to the early onset of a severe form of colitis. GlcNAc supplementation of their diet alleviates disease severity in these mice, thus corroborating that modulating TCR glycosylation could benefit the remediation of colitis. Multiple sclerosis (MS), another inflammatory disease, may also benefit from GlcNAc supplementation, which enhances *N*-glycan branching. Low levels of serum GlcNAc occur in progressive MS, which is a neurodegenerative disease that is characterized by inflammatory demyelination [235]. The supplementation of GlcNAc to MS mouse models diminishes TLR4 and TLR2 signaling in B cells while also reducing pro-inflammatory T-cell-driven demyelination, and it could thus be a promising and inexpensive treatment for MS [236]. Whereas the above findings suggest that enhancing flux through the salvage HBP may mitigate autoimmunity, its benefit may be limited in other cases. For example, naïve T cells of aging mice have particularly increased *N*-glycan branching, which is associated with impaired immunity [237]. Furthermore, the levels of serum GlcNAc increase with age, and serum GlcNAc synergizes with IL-7 signaling to elevate *N*-glycan branching in human T cells. Thus, more studies are needed to define how manipulating GlcNAc levels could serve to prevent autoimmunity as well as improve immune aging. 

The salvage metabolite GlcN has immunosuppressive properties as well [238]. In vitro GlcN supplementation promotes apoptosis of activated but not constitutive primary human T cells [239]. In vivo, GlcN also inhibits Th1- and Th17-mediated autoimmune encephalomyelitis (EAE) in a mouse model of multiple sclerosis [240]. GlcN downregulates *N*-linked glycosylation of the IL2R subunit CD25 and inhibits its downstream signaling [241]. Moreover, high amounts of GlcN reduce *N*-glycosylation of Glut1 in Th1-polarized CD4 T cells, thus contributing to the inhibition of their differentiation. GlcN also inhibits the Th2 immune response in atopic dermatitis-like skin lesions in mice [242]. A combination treatment of GlcN and a low dose of the immunosuppressant cyclosporin A has anti-inflammatory effects on a mouse model of psoriasis [243]. How GlcN affects flux through the HBP and how it could have immunosuppressive effects are unclear. It likely reprograms metabolism to downregulate the usage of glucose (see Section 3.1).

Studies that have examined the role of *O*-GlcNAcylation in T cells also support the importance of the HBP in immunity. The increased UDP-GlcNAc levels in activated T lymphocytes coincides with elevated *O*-GlcNAcylation of intracellular proteins [244,245]. On one hand, the loss of OGT prevents the renewal of T cell progenitors and the clonal expansion of peripheral T cells while also precluding malignant transformation. On the other hand, the deletion of OGA in mouse hematopoietic stem cells also impairs early thymocyte development [246]. *O*-GlcNAcylation is also required to maintain lineage stability and suppressive function of Treg cells [247]. *O*-GlcNAcylation stabilizes Foxp3 and activates STAT5, which are required for the suppressive effector function of Tregs.

The role of the HBP in B cell development and activation has been studied in the context of *O*-GlcNAcylation and *N*-glycan branching. Early activation of B cells by B cell receptor (BCR) stimulation requires OGT [248]. OGT mediates the *O*-GlcNAcylation of the transcription factors NF-kB and NFAT, which play critical roles in B cell immune responses. Deficiency in OGT results in increased apoptosis of germinal center and memory B cells during an immune response, subsequently reducing antibody production [249]. In contrast, OGA inhibition augments B-cell activation and apoptosis following BCR crosslinking [250]. One of the many proteins that undergo *O*-GlcNAcylation during BCR ligation is the lymphocyte-specific protein-1 (Lsp1), which induces apoptosis via the downregulation of Bcl-2 and Bcl-xL. Specific B cell deletion of Mgat1, the key enzyme of *N*-glycan branching, revealed that glycan maturation increases the surface expression of the pre-BCR/BCR co-receptor CD19, thus enhancing low affinity BCR interactions and promoting the positive selection of B cells. At the same time, *N*-glycan branching subdues negative selection by diminishing the induction of Nur77, a marker of negative selection. There is considerable interest in engineering antibody glycosylation due to the many therapeutic applications of antibodies in diseases [251,252]. How modulating the HBP could affect antibody generation and glycosylation would be important to pursue. 

Some studies have unraveled the role of the HBP in innate immune cells such as macrophages and natural killer T (NKT) cells. The LPS-mediated increase in protein *O*-GlcNAcylation occurs in macrophages via the induction of GFAT1 [46]. This elevated *O*-GlcNAcylation attenuates the inflammatory responses triggered by LPS. The HBP could also play a role in the polarization of macrophages, wherein LPS triggers M1 macrophages through Toll-like receptor ligands, whereas IL4 or IL13 trigger alternatively polarized M2 macrophages. Transcriptional and metabolic profiling of macrophage cell lines indicate that UDP-GlcNAc-associated factors are activated during M2 but not M1 polarization [253]. In contrast, the HBP seems to be important in both polarization states of primary macrophages derived from mice bone marrow [254]. Activated NK T cells (NKT) rely on glutamine and utilize the nitrogen of glutamine for hexosamine biosynthesis [255]. Inhibition of the HBP by using DON or blocking OGT reduces protein *O*-GlcNAcylation, resulting in decreased survival, proliferation, and activation of NKT cells.

### 5.4. Cardiac Hypertrophy

Maintaining optimal flux through the HBP is critical for normal cardiac function. Conditions that trigger cardiac stress, such as ischemia/reperfusion, increase the HBP, which serves to protect the heart under stress conditions [39]. Such stress conditions enhance the UPR, which then induces the Xbp1s-mediated expression of GFAT1. This response is associated with cardioprotection. However, chronic upregulation of the HBP can lead to pathological conditions such as cardiac hypertrophy. Increased expression of GFAT1 intensifies pressure-overload-induced cardiac hypertrophy, while its inhibition prevents cardiac hypertrophy induced by phenylephrine, another hypertrophic stimulus [256]. Sustained upregulation of the HBP via GFAT1 overexpression leads to persistent mTOR activation that then triggers decompensated cardiac hypertrophy. AMPK activation prevents cardiac hypertrophy by increasing GFAT1 phosphorylation at Ser243 and diminishing OGT protein levels and *O*-GlcNAcylation [34]. GFAT2 expression in the heart is also increased in response to several hypertrophic stimuli such as isoproterenol (ISO) [45]. Knockdown of GFAT2 expression prevents ISO-induced cardiac hypertrophy as well as the *O*-GlcNAcylation and activation of Akt. In contrast, GlcN administration increases protein *O*-GlcNAcylation, Akt activation, and cardiac hypertrophy. These findings suggest that inhibiting the GFAT2-Akt axis could have a therapeutic benefit during cardiac hypertrophy. The targets of *O*-GlcNAcylation that promote cardiac hypertrophy remain to be characterized, although troponin T could potentially be *O*-GlcNAcylated [34]. Hyperglycemic conditions, which increase flux through the HBP in cardiomyoblasts, increase *O*-GlcNAcylation of the pro-apoptotic protein BAD and augment ROS levels. Increased cellular apoptosis occurs under these conditions [257]. Other substrates that utilize UDP-GlcNAc and play a role in cardiac hypertrophy would need to be further investigated.

### 5.5. Congenital Myasthenic Syndromes

Thirteen unrelated families with autosomal recessive congenital myasthenic syndromes (CMS) have been described to have eighteen different biallelic mutations of *Gfpt1* [258]. Furthermore, over 30 different mutations including missense, nonsense, and frameshift mutations, as well as intronic mutations that affect splicing and mutation in the 3′untranslated region of *Gfpt1,* have been reported to occur in CMS [259,260,261,262]. CMS is characterized by fatigable muscle weakness due to defective neurotransmission. GFAT1-related CMS is also referred to as limb-girdle myasthenia with tubular aggregates (TA). TA are derived from the sarcoplasmic reticulum, forming packed membranous tubules. *N*-glycome analysis of patients with *Gfpt1* mutations reveals no significant defects in global *N*-linked glycosylation relative to normal or CMS patients defined by other mutations [263]. Studies in model organisms are underway to define the pathological mechanisms of this disease. Notably, downregulating GFAT1 alters muscle fiber morphology and impairs the development of the neuromuscular junction (NMJ) in zebrafish [258]. Furthermore, *Gfpt1* mutations or decreased expression of GFAT1 are linked to the diminished expression of cell-surface acetylcholine receptors (AChR) [264]. While the fundamental mechanisms involved in the requirement for GFAT1 or the HBP in CMS remain to be elucidated, so far it is known that *N*-glycosylation of AChR subunits is required for their proper folding and the expression of functional receptors in the plasma membrane [265]. Abrogating *Gfpt1* specifically in mouse skeletal muscle mimics the morphological NMJ changes, fatigable muscle weakness, and progressive myopathy with TA seen in human CMS [266]. The loss of GFAT1 increases the skeletal muscle protein glypican-1, which is involved in NMJ differentiation and maintenance. Increased glypican-1 impairs trafficking of AChRs to and from the endplate. The precise underlying mechanisms involved in *Gfpt1*-mutated CMS as well as whether supplementation with salvage metabolites could have benefits for disease treatment warrant further scrutiny. 

### 5.6. Other Genetic Disorders

Mutations in GNPNAT1 are associated with spondylo-epi-metaphyseal dysplasia (SEMD), a skeletal dysplasia that is characterized by short stature and abnormal bone development in patients [267,268]. Cellular studies suggest that GNPNAT1 is involved in the proliferation and differentiation of growth plate chondrocytes [267]. Mutations in PGM3 also result in skeletal abnormalities resembling Desbuqois dysplasia, which is accompanied by short stature, brachydactyly, dysmorphic facial features, and intellectual disability [269]. These patients additionally have congenital leukopenia and B and T cell lymphopenia, which can progress to bone marrow failure. Mutations in PGM3 have also been associated with a genetic syndrome of severe atopy, autoimmunity, and motor neurocognitive impairment [270]. Patients with PGM3 mutations display reduced levels of UDP-GlcNAc as well as decreased *O*- and *N*-linked protein glycosylation. UAP1 could play a role in human male infertility. Indeed, AGX1 (aka UAP1) is abundantly expressed in the testes of infertile males and implicated in antibody-mediated human infertility [77]. A missense mutation, UAP1-A229T, also exists in a patient with cranial, skeletal, and nervous system abnormalities. This pathogenic mutant renders UAP1 unstable and with decreased activity [271]. It is worth noting that a broad spectrum of diseases termed congenital disorders of glycosylation (CDG), which refers to a family of more than 130 genetic diseases related to mutations in genes involved in *N*- and *O*-glycosylation, shares some similar developmental impairments as those disorders with mutations in enzymes of the HBP. CDG is characterized by developmental delays, hypotonia, neurologic impairments, facial dysmorphisms, and defects in the gastrointestinal tract and the immune system [272]. Mutations in genes involved in several steps along the pathways of glycan modification occur in CDG, such as the phosphomannomutase 2 gene (PMM2), the product of which is involved in the synthesis of GDP-mannose. Together, these findings emphasize how the HBP and glycosylation play crucial developmental roles. Understanding the specific targets of glycosylation in these genetic diseases would shed light on treatment strategies for their underlying defects.

## 6. Manipulating the HBP

**Figure 7:**
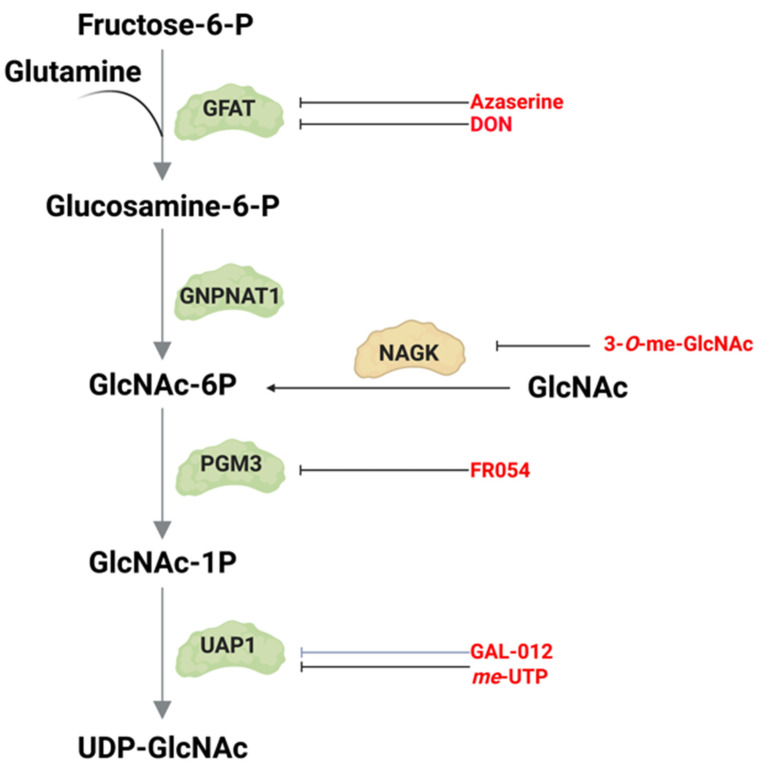
Inhibitors of HBP enzymes.

In addition to the salvage metabolites GlcN and GlcNAc, which are used to modulate the flux through the HBP, pharmacological agents or anti-metabolites have been used to alter HBP activity and/or substrate glycosylation for research purposes or therapeutic benefit (Figure 7). Since GFAT1 catalyzes the key step in de novo HBP, most studies have used inhibitors of this enzyme, which are mainly glutamine analogs. However, such analogs are not highly specific GFAT1 inhibitors since glutamine is also used by other aminotransferases. In addition to hexosamine synthesis, glutamine analogs also inhibit de novo purine and pyrimidine as well as coenzyme and amino acid synthesis [273]. Azaserine, which is isolated from Streptomyces, reacts irreversibly with the N-terminal cysteine residue situated in the glutaminase domain [274,275]. Furthermore, 6-diazo-5-oxo-*L*-norleucine (DON), which is also isolated from Streptomyces, competitively binds to the glutamine active site and is a potent blocker of aminotransferases [22]. These inhibitors have been used in cancer treatment, although early studies revealed undesirable toxicities [273,276]. Recent progress in the understanding of cancer metabolism could forge the development of better strategies and more personalized approaches to enhance efficacy while minimizing toxicity. Early pre-clinical studies have demonstrated the efficacy of glutamine analogs in preventing cancer cell growth in vitro and in xenograft tumors [277,278,279,280,281]. There are also promising results in reducing tumor burden in mice and humans when used alone or in combination with other chemotherapy or immunotherapy [154,159,282,283,284]. Among unwanted side effects, the inhibition of the HBP could also affect the tumor microenvironment. It could also destabilize the expression of checkpoint blockade targets such as the programmed death ligand 1 (PD-L1), a cell surface protein expressed in cancer cells that suppresses T cell activity by binding to the PD1 on T cells. PD-L1 is highly glycosylated, and downregulation of its expression enhances anti-tumor activity of immune cells [285]. Whether the effects of these glutamine analogs are primarily due to targeting GFAT1 remains to be scrutinized.

A series of DON prodrugs has been recently developed to target cancer cells more directly, aiming to temper some of the toxic effects previously caused by DON. JHU083 (ethyl 2-(2-amino-4-methylpentanamido)-DON) was originally developed to improve the delivery of DON to targeted tissues such as the brain and specific tumors [273,286]. By altering certain chemical properties, the prodrug is preferentially bioactivated in the target tissues. Besides tissue-targeting, such strategies allow lower dosage as well as enhanced stability and, more importantly, less overall toxicity. For example, Prodrug 2 (DON) is cleaved/activated more specifically in tumors with overexpression of HDAC and Cathepsin L [273,287]. There are also ongoing efforts to design more specific drugs to target GFAT1 [284,288,289]. 

PGM3 is inhibited by FR054, a prodrug that competes with the PGM3 substrate GlcNAc-6-P [165]. This inhibitor suppresses the growth of breast, lung, and pancreatic cancers in both in vitro and in vivo models [164,165,290,291].

UAP1 inhibitors are also under development [176,177]. A mechanism-inspired UTP α,β-methylenebisphosphonate analog (*me*UTP) has been designed and synthesized and shown to inhibit UAP1 in the micromolar range [177]. By using structure-based virtual screening, a drug-like chemical fragment, GAL-012 was found to inhibit a small family of UDP-hexose pyrophosphorylases including UAP1/AGX1 [176]. While this compound inhibits the growth of cells in vitro, its effect on the HBP remains obscure. Future studies should reveal how UAP1 inhibitors could have beneficial effects in vivo.

The salvage enzyme NAGK is inhibited by 3-*O*-methyl-*N*-acetyl-D-glucosamine (3-*O*-me-GlcNAc) [292]. This inhibitor blocks NAGK as well as *N*-acetyl-mannosamine kinase in liver homogenates and hepatoma cells [293]. How in vivo inhibition of NAGK could be useful to prevent the synthesis of UDP-GlcNAc via the salvage pathway remains to be explored.

Other compounds that affect the metabolism in general or substrate glycosylation in particular may also alter flux through the HBP. Tunicamycin (TM) blocks protein *N*-glycosylation and triggers the UPR by inhibiting DPAGT1 (UDP-glucans: dolichol phosphate *N*-acetylglucosaminephosphotransferase 1), the enzyme that transfers UDP-GlcNAc to the dolichol phosphate that is used for *N*-linked glycosylation [294]. HBP activation and the resulting increased levels of UDP-GlcNAc overcome the toxicity to TM [124]. Furthermore, OGT inhibitors, such as alloxan, could downregulate the flux through the HBP [295]. In contrast, blocking OGA, such as by using PUGNAC, may not upregulate the HBP in pancreatic β-cells. While enhancing the HBP in these cells has adverse effects on their function, PUGNAC (which would sustain protein *O*-GlcNAcylation) does not lead to β-cell deterioration [296]. Glycolysis inhibitors such as 2-deoxyglucose (2-DG) modulate both GFAT1 phosphorylation and activity. 2-DG also acutely enhances flux through the HBP to maintain UDP-GlcNAc levels [12]. However, as glucose is depleted, 2-DG diminishes both UDP-GlcNAc generation and substrate glycosylation [12,297]. How these compounds and anti-metabolites could be exploited to rescue abnormalities in diseases involving the HBP would need to be further addressed. 

## 7. Conclusions and Prospects

Several key nutrients and metabolites merge into the HBP, including the glycolysis metabolite fructose-6-P, the amino acid glutamine, the TCA cycle/lipid metabolism intermediate acetyl-CoA, and the nucleotide UTP. The amounts of substrates, such as fructose-6-P, alone do not dictate flux through the HBP, underscoring the tight regulation of this pathway [12,17,32,33,39]. Enhancing flux through the de novo HBP relies on the availability and activity of GFAT, which can be triggered by glucose limitation, energy stress, AMPK activation, and/or increased protein misfolding [5]. Growth factors and mTOR signaling, both of which also regulate GFAT1, modulate the levels of glutamine [16,40]. In turn, the availability of glutamine impacts GFAT1 expression and de novo HBP activity [14,17,40]. Access to glutamine is also affected by other biosynthetic pathways that require this amino acid, such as purine/pyrimidine biosynthesis [298]. Thus, flux through the HBP is coordinated with other metabolic and signaling pathways. Moreover, metabolic needs are distinct among different tissues and cell types within a tissue microenvironment. Hence, future studies will need to address how the HBP is wired to other metabolic pathways under specific cell type and environmental conditions. Findings from such studies will facilitate more specific targeting of the HBP for therapeutic purposes.

How the salvage pathway works independently, antagonistically, or in synergy with de novo HBP would also need to be interrogated. Genetic studies support that salvage mechanisms compensate for the absence of GFAT1, albeit boosting salvage metabolites is not sufficient to restore all defects associated with the loss of GFAT [83]. In highly proliferating cells such as cancer cells, the de novo HBP may be essential to support robust growth and proliferation. However, when plenty of salvage metabolites become available such as from the tumor microenvironment, this could meet the high demand for UDP-GlcNAc synthesis, as long as the salvage mechanisms are active [14,107]. It is noteworthy that glucose and GlcN share the same transporter, suggesting antagonism between glycolysis and salvage HBP. Upregulating the salvage mechanisms may thus reprogram the overall metabolism and consequently influence growth and proliferation. Indeed, GlcN supplementation prolongs the lifespan of worms and mice via dampening glycolysis and likely independently affecting the HBP [87]. Nevertheless, enhancing the HBP also prolongs the lifespan of *C. elegans* due to improved proteostasis. Hence, the role of salvage mechanisms and their relationship with the de novo HBP and other biosynthetic and signaling pathways warrant further interrogation. How amino acid, lipid, and nucleotide metabolism, the products of which have input into the HBP, impact flux through the HBP also needs to be further investigated. 

Understanding the regulation of the HBP should bring more insights on how protein glycosylation is controlled. The *N*-glycan profile and *O*-linked glycosylation of proteins serve as useful biomarkers, diagnostic tools, and therapeutic targets [185,226,299,300]. Given the heterogeneous nature of cell surface and secretory protein *N*-glycan branching and how it affects cell fate and responses [2], more studies should address how flux through the de novo vs salvage pathways affects this process. Likewise, *O*-GlcNAcylation is a prevalent modification occurring in metabolic enzymes, transcriptional and epigenetic regulators, and signaling proteins [130]. Since it is partly dependent on the availability of UDP-GlcNAc, this modification could thus coordinate the HBP flux with other metabolic and signaling pathways as well as gene expression. Unravelling the specific functions of *N*- and *O*-GlcNAcylated proteins and how they become deregulated in disease should also yield insights on viable targets to treat specific disorders. While current compounds that are widely used to inhibit the de novo HBP are highly unspecific, the development of prodrugs aims to minimize toxicity while maximizing specificity [273]. The design of more specific compounds to inhibit HBP enzymes and whether they will have efficacy in disease treatment needs to be explored. Furthermore, given that different cell types could have unique metabolic dependencies, it would be important to probe how we can manipulate the HBP in combination with dietary supplementation/limitation to effectively and specifically target abnormal cells within a tissue microenvironment. Inhibiting the HBP in the target cells could also improve the effects of other therapeutics, for example, immunotherapy [159]. As we broaden our understanding of the HBP and metabolism in general and how they are linked to cell signaling pathways, we should be able to develop more effective therapeutic strategies to treat a variety of diseases with underlying glycosylation or metabolic defects.

## Data Availability

Not applicable.

## Abbreviations

2-DG	2-deoxyglucose
ACE	angiotensin-converting enzyme
AChR	acetylcholine receptor
AGX1 and 2	alanine–glyoxylate aminotransferase 1
AKT/PKB	protein kinase B
AMDHD2	*N*-acetylglucosamine deacetylase
AML	acute myeloid leukemia
AMPK	AMP-activated protein kinase
ATF4	activating transcription factor 4
ATF6	activating transcription factor 6
ATP	adenosine triphosphate
AUF1	AU-rich element RNA-binding protein 1
BAD	BCL2 Associated Agonist of Cell Death
Bcl-xL	B-cell lymphoma-extra large
CD8-ISP	CD8-immature single positive
CDG	congenital disorders of glycosylation
ChREBP	carbohydrate-response element-binding protein
CMS	congenital myasthenic syndrome
CNS	central nervous system
COPII	coat complex II
CRC	colorectal cancer
CRPC	castration-resistant prostate cancer
CSC	cancer stem cells
CTP	cytidine triphosphate
DLBCL	diffuse large B-cell lymphoma
DN	double negative
DN3-DN4	double negative 3 and double negative 4
dNTP	deoxynucleotide triphosphate
DON	6-Diazo-5-oxo-*L*-norleucine
DPAGT1	UDP-GlcNAc: dolichol phosphate *N*-acetylglucosaminephosphotransferase 1
DUF	domain with unknown function
EAE	experimental autoimmune encephalomyelitis
ECM	extracellular matrix
EGFR	epidermal growth factor receptor
eIF2α	eukaryotic initiation factor-2-α
EMT	epithelial–mesenchymal transition
ER	endoplasmic reticulum
ERAD	ER-associated protein degradation
Fbxl17	F-box and leucine rich repeat protein 17
FoxO1	forkhead box O1
Foxp3	forkhead box P3
Frc-6-P	fructose-6-phosphate
GalNAc1P	*N*-acetylgalactosamine-1-phosphate
GCK	glucokinase
GCN2	general control non-depressible 2
GDP-mannose	guanosine diphosphate mannose
GFAT/GFPT 1 and 2	glutamine:fructose-6-phosphate amidotransferase 1 and 2
GlcN-6-P	glucosamine-6-phosphate
GlcN/GAM	glucosamine
GlcNAc-1-P	*N*-acetylglucosamine-1-phosphate
GlcNAc-6-P	*N*-acetylglucosamine-6-phosphate
GlcNAc/NAG	*N*-acetylglucosamine
GLUT1 and 2 and 4	glucose transporter 1 and 2 and 4
GNPDA1 and 2	glucosamine-6-phosphate deaminase 1 and 2
GNPNAT1/GNA1	glucosamine-6-phosphate *N*-acetyltransferase 1
GPI	glycosylphosphatidylinositol
GPT	UDP-GlcNAc:dolichoylphosphate GlcNAc-1-phosphotransferase
GTK	glutamine transaminase K
HA	hyaluronan/hyaluronic acid
HBP	hexosamine biosynthetic pathway
HCC	hepatocellular carcinoma
HIF1α	Hypoxia-inducible factor 1-α
HRE	hypoxia response element
HUVEC	human umbilical vein endothelial cell
IL-13	interleukin 13
IL-4	interleukin 4
IL-7	interleukin 7
IL2R	interleukin-2 receptor
IRE1	Inositol-requiring enzyme 1
IRS-1	insulin receptor substrate 1
ISO	isoproterenol
ISR	integrated stress response
KL NSCLC	keratinizing squamous cell carcinoma of the non-small cell lung cancer
KRAS	Kirsten rat sarcoma virus
LARS1	leucyl-tRNA synthetase 1
LKB1	liver kinase B1
LND	lonidamine
LPS	lipopolysaccharides
LUAD	lung adenocarcinoma
LUSC	lung squamous cell carcinoma
LXR	liver receptor X
MEF	mouse embryonic fibroblasts
mESC	murine embryonic stem cells
MPNST	malignant peripheral nerve sheath tumor
mRNA	messenger RNA
MS	multiple sclerosis
mTOR	mechanistic target of rapamycin
mTORC1 and 2	mTOR complex 1 and 2
MYBL1	MYB proto-oncogene-like 1
NAGK	*N*-acetylglucosamine kinase
NF-κB	nuclear factor kappa B
NK	natural killer
NKT	NK T cells
NR4A1	nuclear receptor subfamily 4 group A member 1
NSCLC	non-small cell lung cancer
OGA	*O*-GlcNAcase
OGT	*O*-GlcNAc transferase
OxPhos	oxidative phosphorylation
PCK1	phosphoenolpyruvate carboxykinase 1
PD1	programmed cell death protein 1
PDAC	pancreatic ductal adenocarcinoma
PDK1	pyruvate dehydrogenase kinase 1
PERK	protein kinase R-like endoplasmic reticulum kinase
PFK1	phosphofructokinase 1
PGC1α	PPARG coactivator 1 α
PGM3	phosphoglucomutase 3
PI3K	phosphoinositide 3-kinases
PKA	protein kinase A
PKM2	pyruvate kinase M2
PKR	double-stranded RNA-activated protein kinase
PMM2	phosphomannomutase 2
polyQ	polyglutamine
PTEN	phosphatase and tensin homolog
REDD1	regulated in development and DNA damage responses 1
RNR	ribonucleotide reductase
ROS	reactive oxygen species
SEMD	spondylo-epi-metaphyseal dysplasia
SIN1	stress-activated protein kinase
SLP-2	stomatin-like protein 2
SP	single positive
Sp1	specificity protein 1
SRPK2	serine-/arginine-rich protein kinase 2
STAT3 and 5	signal transducer and activator of transcription 3 and 5
TA	tubular aggregates
TAB1	transforming growth factor-β-activated kinase-binding protein 1
TCR	T cell receptor
TH	T helper
Th1 and Th2	T helper type 1 and 2
TLR4 and 2	toll-like receptor 4 and 2
TM	tunicamycin
TME	tumor microenvironment
Treg	T regulatory
tRNA	transfer RNA
TTLL5	tubulin-tyrosine ligase-like 5
UAP1	UDP-*N*-acetylhexosamine pyrophosphrylase 1
UC	ulcerative colitis
UDP-GalNAc	uridine diphosphate-*N*-acetyl galactosamine
UDP-GlcNAc	uridine diphosphate-*N*-acetyl glucosamine
UDP-HexNAc	uridine diphosphate *N*-acetyl hexosamine
UPR	unfolded protein response
UTP	uridine triphosphate
VCP	valosin-containing proteins
VEGF	vascular endothelial growth factor
Xbp1s	spliced x-box-binding protein 1

## References

[1] Reily C., Stewart T.J., Renfrow M.B., Novak J. (2019). Glycosylation in health and disease. Nat. Rev. Nephrol..

[2] Dennis J.W., Nabi I.R., Demetriou M. (2009). Metabolism, cell surface organization, and disease. Cell.

[3] Flynn R.A., Pedram K., Malaker S.A., Batista P.J., Smith B.A.H., Johnson A.G., George B.M., Majzoub K., Villalta P.W., Carette J.E. (2021). Small RNAs are modified with N-glycans and displayed on the surface of living cells. Cell.

[4] Thoden J.B., Wohlers T.M., Fridovich-Keil J.L., Holden H.M. (2001). Human UDP-galactose 4-epimerase. Accommodation of UDP-N-acetylglucosamine within the active site. J. Biol. Chem..

[5] Denzel M.S., Antebi A. (2015). Hexosamine pathway and (ER) protein quality control. Curr. Opin. Cell. Biol..

[6] Vasseur S., Manie S.N. (2015). ER stress and hexosamine pathway during tumourigenesis: A pas de deux?. Semin. Cancer Biol..

[7] Wyllie J.A., McKay M.V., Barrow A.S., Soares da Costa T.P. (2022). Biosynthesis of uridine diphosphate N-Acetylglucosamine: An underexploited pathway in the search for novel antibiotics?. IUBMB Life.

[8] Marshall S., Bacote V., Traxinger R.R. (1991). Discovery of a metabolic pathway mediating glucose-induced desensitization of the glucose transport system. Role of hexosamine biosynthesis in the induction of insulin resistance. J. Biol. Chem..

[9] Araujo L., Khim P., Mkhikian H., Mortales C.L., Demetriou M. (2017). Glycolysis and glutaminolysis cooperatively control T cell function by limiting metabolite supply to N-glycosylation. eLife.

[10] Marshall S., Nadeau O., Yamasaki K. (2004). Dynamic actions of glucose and glucosamine on hexosamine biosynthesis in isolated adipocytes: Differential effects on glucosamine 6-phosphate, UDP-N-acetylglucosamine, and ATP levels. J. Biol. Chem..

[11] Bosch R.R., Pouwels M.J., Span P.N., Olthaar A.J., Tack C.J., Hermus A.R., Sweep C.G. (2004). Hexosamines are unlikely to function as a nutrient-sensor in 3T3-L1 adipocytes: A comparison of UDP-hexosamine levels after increased glucose flux and glucosamine treatment. Endocrine.

[12] Moloughney J.G., Vega-Cotto N.M., Liu S., Patel C., Kim P.K., Wu C.C., Albaciete D., Magaway C., Chang A., Rajput S. (2018). mTORC2 modulates the amplitude and duration of GFAT1 Ser-243 phosphorylation to maintain flux through the hexosamine pathway during starvation. J. Biol. Chem..

[13] Olson A.K., Bouchard B., Zhu W.Z., Chatham J.C., Des Rosiers C. (2020). First characterization of glucose flux through the hexosamine biosynthesis pathway (HBP) in ex vivo mouse heart. J. Biol. Chem..

[14] Campbell S., Mesaros C., Izzo L., Affronti H., Noji M., Schaffer B.E., Tsang T., Sun K., Trefely S., Kruijning S. (2021). Glutamine deprivation triggers NAGK-dependent hexosamine salvage. eLife.

[15] Szwed A., Kim E., Jacinto E. (2021). Regulation and metabolic functions of mTORC1 and mTORC2. Physiol. Rev..

[16] Wellen K.E., Lu C., Mancuso A., Lemons J.M., Ryczko M., Dennis J.W., Rabinowitz J.D., Coller H.A., Thompson C.B. (2010). The hexosamine biosynthetic pathway couples growth factor-induced glutamine uptake to glucose metabolism. Genes. Dev..

[17] Chaveroux C., Sarcinelli C., Barbet V., Belfeki S., Barthelaix A., Ferraro-Peyret C., Lebecque S., Renno T., Bruhat A., Fafournoux P. (2016). Nutrient shortage triggers the hexosamine biosynthetic pathway via the GCN2-ATF4 signalling pathway. Sci. Rep..

[18] Oki T., Yamazaki K., Kuromitsu J., Okada M., Tanaka I. (1999). cDNA cloning and mapping of a novel subtype of glutamine:fructose-6-phosphate amidotransferase (GFAT2) in human and mouse. Genomics.

[19] Nabeebaccus A.A., Verma S., Zoccarato A., Emanuelli G., Santos C.X., Streckfuss-Bomeke K., Shah A.M. (2021). Cardiomyocyte protein O-GlcNAcylation is regulated by GFAT1 not GFAT2. Biochem. Biophys. Res. Commun..

[20] Niimi M., Ogawara T., Yamashita T., Yamamoto Y., Ueyama A., Kambe T., Okamoto T., Ban T., Tamanoi H., Ozaki K. (2001). Identification of GFAT1-L, a novel splice variant of human glutamine: Fructose-6-phosphate amidotransferase (GFAT1) that is expressed abundantly in skeletal muscle. J. Hum. Genet..

[21] DeHaven J.E., Robinson K.A., Nelson B.A., Buse M.G. (2001). A novel variant of glutamine: Fructose-6-phosphate amidotransferase-1 (GFAT1) mRNA is selectively expressed in striated muscle. Diabetes.

[22] Ghosh S., Blumenthal H.J., Davidson E., Roseman S. (1960). Glucosamine metabolism. V. Enzymatic synthesis of glucosamine 6-phosphate. J. Biol. Chem..

[23] Floquet N., Mouilleron S., Daher R., Maigret B., Badet B., Badet-Denisot M.A. (2007). Ammonia channeling in bacterial glucosamine-6-phosphate synthase (Glms): Molecular dynamics simulations and kinetic studies of protein mutants. FEBS Lett..

[24] Mouilleron S., Badet-Denisot M.A., Golinelli-Pimpaneau B. (2008). Ordering of C-terminal loop and glutaminase domains of glucosamine-6-phosphate synthase promotes sugar ring opening and formation of the ammonia channel. J. Mol. Biol..

[25] Broschat K.O., Gorka C., Page J.D., Martin-Berger C.L., Davies M.S., Huang Hc H.C., Gulve E.A., Salsgiver W.J., Kasten T.P. (2002). Kinetic characterization of human glutamine-fructose-6-phosphate amidotransferase I: Potent feedback inhibition by glucosamine 6-phosphate. J. Biol. Chem..

[26] Ruegenberg S., Horn M., Pichlo C., Allmeroth K., Baumann U., Denzel M.S. (2020). Loss of GFAT-1 feedback regulation activates the hexosamine pathway that modulates protein homeostasis. Nat. Commun..

[27] Hu Y., Riesland L., Paterson A.J., Kudlow J.E. (2004). Phosphorylation of mouse glutamine-fructose-6-phosphate amidotransferase 2 (GFAT2) by cAMP-dependent protein kinase increases the enzyme activity. J. Biol. Chem..

[28] Zhou J., Huynh Q.K., Hoffman R.T., Crook E.D., Daniels M.C., Gulve E.A., McClain D.A. (1998). Regulation of glutamine:fructose-6-phosphate amidotransferase by cAMP-dependent protein kinase. Diabetes.

[29] Chang Q., Su K., Baker J.R., Yang X., Paterson A.J., Kudlow J.E. (2000). Phosphorylation of human glutamine:fructose-6-phosphate amidotransferase by cAMP-dependent protein kinase at serine 205 blocks the enzyme activity. J. Biol. Chem..

[30] Ruegenberg S., Mayr F., Atanassov I., Baumann U., Denzel M.S. (2021). Protein kinase A controls the hexosamine pathway by tuning the feedback inhibition of GFAT-1. Nat. Commun..

[31] Yang H., Yang L. (2016). Targeting cAMP/PKA pathway for glycemic control and type 2 diabetes therapy. J. Mol. Endocrinol..

[32] Eguchi S., Oshiro N., Miyamoto T., Yoshino K., Okamoto S., Ono T., Kikkawa U., Yonezawa K. (2009). AMP-activated protein kinase phosphorylates glutamine: Fructose-6-phosphate amidotransferase 1 at Ser243 to modulate its enzymatic activity. Genes. Cells.

[33] Zibrova D., Vandermoere F., Goransson O., Peggie M., Marino K.V., Knierim A., Spengler K., Weigert C., Viollet B., Morrice N.A. (2017). GFAT1 phosphorylation by AMPK promotes VEGF-induced angiogenesis. Biochem. J..

[34] Gelinas R., Mailleux F., Dontaine J., Bultot L., Demeulder B., Ginion A., Daskalopoulos E.P., Esfahani H., Dubois-Deruy E., Lauzier B. (2018). AMPK activation counteracts cardiac hypertrophy by reducing O-GlcNAcylation. Nat. Commun..

[35] Li M.L., Ragupathi A., Patel N., Hernandez T., Magsino J., Werlen G., Brewer G., Jacinto E. (2022). The RNA-binding protein AUF1 facilitates Akt phosphorylation at the membrane. J. Biol. Chem..

[36] Sayeski P.P., Wang D., Su K., Han I.O., Kudlow J.E. (1997). Cloning and partial characterization of the mouse glutamine:fructose-6-phosphate amidotransferase (GFAT) gene promoter. Nucleic Acids Res..

[37] Girard J., Ferre P., Foufelle F. (1997). Mechanisms by which carbohydrates regulate expression of genes for glycolytic and lipogenic enzymes. Annu. Rev. Nutr..

[38] Yang X., Su K., Roos M.D., Chang Q., Paterson A.J., Kudlow J.E. (2001). O-linkage of N-acetylglucosamine to Sp1 activation domain inhibits its transcriptional capability. Proc. Natl. Acad. Sci. USA.

[39] Wang Z.V., Deng Y., Gao N., Pedrozo Z., Li D.L., Morales C.R., Criollo A., Luo X., Tan W., Jiang N. (2014). Spliced X-box binding protein 1 couples the unfolded protein response to hexosamine biosynthetic pathway. Cell.

[40] Moloughney J.G., Kim P.K., Vega-Cotto N.M., Wu C.C., Zhang S., Adlam M., Lynch T., Chou P.C., Rabinowitz J.D., Werlen G. (2016). mTORC2 Responds to Glutamine Catabolite Levels to Modulate the Hexosamine Biosynthesis Enzyme GFAT1. Mol. Cell..

[41] Manzari B., Kudlow J.E., Fardin P., Merello E., Ottaviano C., Puppo M., Eva A., Varesio L. (2007). Induction of macrophage glutamine: Fructose-6-phosphate amidotransferase expression by hypoxia and by picolinic acid. Int. J. Immunopathol. Pharm..

[42] Li D., Guan M., Cao X., Qiang Zha Z., Zhang P., Xiang H., Zhou Y., Peng Q., Xu Z., Lu L. (2022). GFPT1 promotes the proliferation of cervical cancer via regulating the ubiquitination and degradation of PTEN. Carcinogenesis.

[43] Wei S., Zhao Q., Zheng K., Liu P., Sha N., Li Y., Ma C., Li J., Zhuo L., Liu G. (2022). GFAT1-linked TAB1 glutamylation sustains p38 MAPK activation and promotes lung cancer cell survival under glucose starvation. Cell. Discov..

[44] Kroef V., Ruegenberg S., Horn M., Allmeroth K., Ebert L., Bozkus S., Miethe S., Elling U., Schermer B., Baumann U. (2022). GFPT2/GFAT2 and AMDHD2 act in tandem to control the hexosamine pathway. eLife.

[45] Ishikita A., Matsushima S., Ikeda S., Okabe K., Nishimura R., Tadokoro T., Enzan N., Yamamoto T., Sada M., Tsutsui Y. (2021). GFAT2 mediates cardiac hypertrophy through HBP-O-GlcNAcylation-Akt pathway. iScience.

[46] Al-Mukh H., Baudoin L., Bouaboud A., Sanchez-Salgado J.L., Maraqa N., Khair M., Pagesy P., Bismuth G., Niedergang F., Issad T. (2020). Lipopolysaccharide Induces GFAT2 Expression to Promote O-Linked β-N-Acetylglucosaminylation and Attenuate Inflammation in Macrophages. J. Immunol..

[47] Dai W., Dierschke S.K., Toro A.L., Dennis M.D. (2018). Consumption of a high fat diet promotes protein O-GlcNAcylation in mouse retina via NR4A1-dependent GFAT2 expression. Biochim. Biophys. Acta Mol. Basis Dis..

[48] Zitzler J., Link D., Schafer R., Liebetrau W., Kazinski M., Bonin-Debs A., Behl C., Buckel P., Brinkmann U. (2004). High-throughput functional genomics identifies genes that ameliorate toxicity due to oxidative stress in neuronal HT-22 cells: GFPT2 protects cells against peroxide. Mol. Cell. Proteom..

[49] Oikari S., Kettunen T., Tiainen S., Hayrinen J., Masarwah A., Sudah M., Sutela A., Vanninen R., Tammi M., Auvinen P. (2018). UDP-sugar accumulation drives hyaluronan synthesis in breast cancer. Matrix Biol..

[50] Leung D., Price Z.K., Lokman N.A., Wang W., Goonetilleke L., Kadife E., Oehler M.K., Ricciardelli C., Kannourakis G., Ahmed N. (2022). Platinum-resistance in epithelial ovarian cancer: An interplay of epithelial-mesenchymal transition interlinked with reprogrammed metabolism. J. Transl. Med..

[51] Ding X., Liu H., Yuan Y., Zhong Q., Zhong X. (2022). Roles of GFPT2 Expression Levels on the Prognosis and Tumor Microenvironment of Colon Cancer. Front. Oncol..

[52] Tolwani A., Matusiak M., Bui N., Forgo E., Varma S., Baratto L., Iagaru A., Lazar A.J., van de Rijn M., Przybyl J. (2021). Prognostic relevance of the hexosamine biosynthesis pathway activation in leiomyosarcoma. NPJ Genom. Med..

[53] Szymura S.J., Zaemes J.P., Allison D.F., Clift S.H., D’Innocenzi J.M., Gray L.G., McKenna B.D., Morris B.B., Bekiranov S., LeGallo R.D. (2019). NF-kappaB upregulates glutamine-fructose-6-phosphate transaminase 2 to promote migration in non-small cell lung cancer. Cell. Commun. Signal..

[54] Li J., Ye Y., Liu Z., Zhang G., Dai H., Li J., Zhou B., Li Y., Zhao Q., Huang J. (2022). Macrophage mitochondrial fission improves cancer cell phagocytosis induced by therapeutic antibodies and is impaired by glutamine competition. Nat. Cancer.

[55] Oliveira I.A., Allonso D., Fernandes T.V.A., Lucena D.M.S., Ventura G.T., Dias W.B., Mohana-Borges R.S., Pascutti P.G., Todeschini A.R. (2021). Enzymatic and structural properties of human glutamine:fructose-6-phosphate amidotransferase 2 (hGFAT2). J. Biol. Chem..

[56] Kornfeld R. (1967). Studies on L-glutamine D-fructose 6-phosphate amidotransferase. I. Feedback inhibition by uridine diphosphate-N-acetylglucosamine. J. Biol. Chem..

[57] Richez C., Boetzel J., Floquet N., Koteshwar K., Stevens J., Badet B., Badet-Denisot M.A. (2007). Expression and purification of active human internal His(6)-tagged L-glutamine: D-Fructose-6P amidotransferase I. Protein Expr. Purif..

[58] Yamazaki K., Mizui Y., Oki T., Okada M., Tanaka I. (2000). Cloning and characterization of mouse glutamine:fructose-6-phosphate amidotransferase 2 gene promoter. Gene.

[59] Chao D., Ariake K., Sato S., Ohtsuka H., Takadate T., Ishida M., Masuda K., Maeda S., Miura T., Mitachi K. (2021). Stomatinlike protein 2 induces metastasis by regulating the expression of a ratelimiting enzyme of the hexosamine biosynthetic pathway in pancreatic cancer. Oncol. Rep..

[60] Wang J., Liu X., Liang Y.H., Li L.F., Su X.D. (2008). Acceptor substrate binding revealed by crystal structure of human glucosamine-6-phosphate N-acetyltransferase 1. FEBS Lett..

[61] Boehmelt G., Wakeham A., Elia A., Sasaki T., Plyte S., Potter J., Yang Y., Tsang E., Ruland J., Iscove N.N. (2000). Decreased UDP-GlcNAc levels abrogate proliferation control in EMeg32-deficient cells. EMBO J..

[62] Ding P., Peng B., Li G., Sun X., Wang G. (2022). Glucosamine-phosphate N-acetyltransferase 1 and its DNA methylation can be biomarkers for the diagnosis and prognosis of lung cancer. J. Clin. Lab. Anal..

[63] Meng J., Cao L., Song H., Chen L., Qu Z. (2021). Integrated analysis of gene expression and DNA methylation datasets identified key genes and a 6-gene prognostic signature for primary lung adenocarcinoma. Genet. Mol. Biol..

[64] Bacos K., Gillberg L., Volkov P., Olsson A.H., Hansen T., Pedersen O., Gjesing A.P., Eiberg H., Tuomi T., Almgren P. (2016). Blood-based biomarkers of age-associated epigenetic changes in human islets associate with insulin secretion and diabetes. Nat. Commun..

[65] Arreola R., Valderrama B., Morante M.L., Horjales E. (2003). Two mammalian glucosamine-6-phosphate deaminases: A structural and genetic study. FEBS Lett..

[66] Oikari S., Makkonen K., Deen A.J., Tyni I., Karna R., Tammi R.H., Tammi M.I. (2016). Hexosamine biosynthesis in keratinocytes: Roles of GFAT and GNPDA enzymes in the maintenance of UDP-GlcNAc content and hyaluronan synthesis. Glycobiology.

[67] Willer C.J., Speliotes E.K., Loos R.J., Li S., Lindgren C.M., Heid I.M., Berndt S.I., Elliott A.L., Jackson A.U., Lamina C. (2009). Six new loci associated with body mass index highlight a neuronal influence on body weight regulation. Nat. Genet..

[68] Gutierrez-Aguilar R., Grayson B.E., Kim D.H., Yalamanchili S., Calcagno M.L., Woods S.C., Seeley R.J. (2021). CNS GNPDA2 Does Not Control Appetite, but Regulates Glucose Homeostasis. Front. Nutr..

[69] Wu L., Ma F., Zhao X., Zhang M.X., Wu J., Mi J. (2019). GNPDA2 Gene Affects Adipogenesis and Alters the Transcriptome Profile of Human Adipose-Derived Mesenchymal Stem Cells. Int. J. Endocrinol..

[70] Yoganathan P., Karunakaran S., Ho M.M., Clee S.M. (2012). Nutritional regulation of genome-wide association obesity genes in a tissue-dependent manner. Nutr. Metab..

[71] Jolly L., Ferrari P., Blanot D., Van Heijenoort J., Fassy F., Mengin-Lecreulx D. (1999). Reaction mechanism of phosphoglucosamine mutase from *Escherichia coli*. Eur. J. Biochem..

[72] Mio T., Yamada-Okabe T., Arisawa M., Yamada-Okabe H. (2000). Functional cloning and mutational analysis of the human cDNA for phosphoacetylglucosamine mutase: Identification of the amino acid residues essential for the catalysis. Biochim. Biophys. Acta.

[73] Hopkinson D.A., Harris H. (1968). A third phosphoglucomutase locus in man. Ann. Hum. Genet..

[74] Greig K.T., Antonchuk J., Metcalf D., Morgan P.O., Krebs D.L., Zhang J.G., Hacking D.F., Bode L., Robb L., Kranz C. (2007). Agm1/Pgm3-mediated sugar nucleotide synthesis is essential for hematopoiesis and development. Mol. Cell. Biol..

[75] Weidanz J.A., Campbell P., Moore D., DeLucas L.J., Roden L., Thompson J.N., Vezza A.C. (1996). N-acetylglucosamine kinase and N-acetylglucosamine 6-phosphate deacetylase in normal human erythrocytes and Plasmodium falciparum. Br. J. Haematol..

[76] Mio T., Yabe T., Arisawa M., Yamada-Okabe H. (1998). The eukaryotic UDP-N-acetylglucosamine pyrophosphorylases. Gene cloning, protein expression, and catalytic mechanism. J. Biol. Chem..

[77] Diekman A.B., Goldberg E. (1994). Characterization of a human antigen with sera from infertile patients. Biol. Reprod..

[78] Wang-Gillam A., Pastuszak I., Elbein A.D. (1998). A 17-amino acid insert changes UDP-N-acetylhexosamine pyrophosphorylase specificity from UDP-GalNAc to UDP-GlcNAc. J. Biol. Chem..

[79] Peneff C., Ferrari P., Charrier V., Taburet Y., Monnier C., Zamboni V., Winter J., Harnois M., Fassy F., Bourne Y. (2001). Crystal structures of two human pyrophosphorylase isoforms in complexes with UDPGlc(Gal)NAc: Role of the alternatively spliced insert in the enzyme oligomeric assembly and active site architecture. EMBO J..

[80] Mason B., Flach S., Teixeira F.R., Manzano Garcia R., Rueda O.M., Abraham J.E., Caldas C., Edwards P.A.W., Laman H. (2020). Fbxl17 is rearranged in breast cancer and loss of its activity leads to increased global O-GlcNAcylation. Cell. Mol. Life Sci..

[81] Kornfeld S., Ginsburg V. (1966). The metabolism of glucosamine by tissue culture cells. Exp. Cell. Res..

[82] Krug E., Zweibaum A., Schulz-Holstege C., Keppler D. (1984). D-glucosamine-induced changes in nucleotide metabolism and growth of colon-carcinoma cells in culture. Biochem. J..

[83] Werlen G., Li M.L., Tottone L., da Silva-Diz V., Su X., Herranz D., Jacinto E. (2022). Dietary glucosamine overcomes the defects in alphabeta-T cell ontogeny caused by the loss of de novo hexosamine biosynthesis. Nat. Commun..

[84] Uldry M., Ibberson M., Hosokawa M., Thorens B. (2002). GLUT2 is a high affinity glucosamine transporter. FEBS Lett..

[85] Marshall S., Yamasaki K., Okuyama R. (2005). Glucosamine induces rapid desensitization of glucose transport in isolated adipocytes by increasing GlcN-6-P levels. Biochem. Biophys. Res. Commun..

[86] Silverman J.L. (1963). Glucosamine Inhibition of (I-14c)Glucose Oxidation as Measured by Rat Adipose Tissue in Vitro. Biochim. Biophys. Acta.

[87] Weimer S., Priebs J., Kuhlow D., Groth M., Priebe S., Mansfeld J., Merry T.L., Dubuis S., Laube B., Pfeiffer A.F. (2014). D-Glucosamine supplementation extends life span of nematodes and of ageing mice. Nat. Commun..

[88] Moore J.A., Miller W.P., Dennis M.D. (2016). Glucosamine induces REDD1 to suppress insulin action in retinal Muller cells. Cell. Signal..

[89] Robinson K.A., Sens D.A., Buse M.G. (1993). Pre-exposure to glucosamine induces insulin resistance of glucose transport and glycogen synthesis in isolated rat skeletal muscles. Study of mechanisms in muscle and in rat-1 fibroblasts overexpressing the human insulin receptor. Diabetes.

[90] Patti M.E., Virkamaki A., Landaker E.J., Kahn C.R., Yki-Jarvinen H. (1999). Activation of the hexosamine pathway by glucosamine in vivo induces insulin resistance of early postreceptor insulin signaling events in skeletal muscle. Diabetes.

[91] Monauni T., Zenti M.G., Cretti A., Daniels M.C., Targher G., Caruso B., Caputo M., McClain D., Del Prato S., Giaccari A. (2000). Effects of glucosamine infusion on insulin secretion and insulin action in humans. Diabetes.

[92] Simon R.R., Marks V., Leeds A.R., Anderson J.W. (2011). A comprehensive review of oral glucosamine use and effects on glucose metabolism in normal and diabetic individuals. Diabetes Metab. Res. Rev..

[93] Clegg D.O., Reda D.J., Harris C.L., Klein M.A., O’Dell J.R., Hooper M.M., Bradley J.D., Bingham C.O., Weisman M.H., Jackson C.G. (2006). Glucosamine, chondroitin sulfate, and the two in combination for painful knee osteoarthritis. N. Engl. J. Med..

[94] Shintani T., Kosuge Y., Ashida H. (2018). Glucosamine Extends the Lifespan of *Caenorhabditis elegans* via Autophagy Induction. J. Appl. Glycosci..

[95] Zhou J., Wu Z., Lin Z., Wang W., Wan R., Liu T. (2022). Association between glucosamine use and cancer mortality: A large prospective cohort study. Front. Nutr..

[96] Jung C.W., Jo J.R., Lee S.H., Park Y.K., Jung N.K., Song D.K., Bae J., Nam K.Y., Ha J.S., Park I.S. (2012). Anti-cancer properties of glucosamine-hydrochloride in YD-8 human oral cancer cells: Induction of the caspase-dependent apoptosis and down-regulation of HIF-1alpha. Toxicol. Vitr..

[97] Wang L.S., Chen S.J., Zhang J.F., Liu M.N., Zheng J.H., Yao X.D. (2017). Anti-proliferative potential of Glucosamine in renal cancer cells via inducing cell cycle arrest at G0/G1 phase. BMC Urol..

[98] Quastel J.H., Cantero A. (1953). Inhibition of tumour growth by D-glucosamine. Nature.

[99] Chesnokov V., Sun C., Itakura K. (2009). Glucosamine suppresses proliferation of human prostate carcinoma DU145 cells through inhibition of STAT3 signaling. Cancer Cell. Int..

[100] Wang Z., Liang R., Huang G.S., Piao Y., Zhang Y.Q., Wang A.Q., Dong B.X., Feng J.L., Yang G.R., Guo Y. (2006). Glucosamine sulfate-induced apoptosis in chronic myelogenous leukemia K562 cells is associated with translocation of cathepsin D and downregulation of Bcl-xL. Apoptosis.

[101] Li G., Zhang X., Liu Y., Zhang J., Li L., Huang X., Thabane L., Lip G.Y.H. (2022). Relationship between glucosamine use and the risk of lung cancer: Data from a nationwide prospective cohort study. Eur. Respir. J..

[102] Berger M., Chen H., Reutter W., Hinderlich S. (2002). Structure and function of N-acetylglucosamine kinase. Identification of two active site cysteines. Eur. J. Biochem..

[103] Ryczko M.C., Pawling J., Chen R., Abdel Rahman A.M., Yau K., Copeland J.K., Zhang C., Surendra A., Guttman D.S., Figeys D. (2016). Metabolic Reprogramming by Hexosamine Biosynthetic and Golgi N-Glycan Branching Pathways. Sci. Rep..

[104] Dickinson M.E., Flenniken A.M., Ji X., Teboul L., Wong M.D., White J.K., Meehan T.F., Weninger W.J., Westerberg H., Adissu H. (2016). High-throughput discovery of novel developmental phenotypes. Nature.

[105] Neitzel L.R., Spencer Z.T., Nayak A., Cselenyi C.S., Benchabane H., Youngblood C.Q., Zouaoui A., Ng V., Stephens L., Hann T. (2019). Developmental regulation of Wnt signaling by Nagk and the UDP-GlcNAc salvage pathway. Mech. Dev..

[106] Maguire P.B., Wynne K.J., Harney D.F., O’Donoghue N.M., Stephens G., Fitzgerald D.J. (2002). Identification of the phosphotyrosine proteome from thrombin activated platelets. Proteomics.

[107] Kim P.K., Halbrook C.J., Kerk S.A., Radyk M., Wisner S., Kremer D.M., Sajjakulnukit P., Andren A., Hou S.W., Trivedi A. (2021). Hyaluronic acid fuels pancreatic cancer cell growth. eLife.

[108] Wolf A.J., Reyes C.N., Liang W., Becker C., Shimada K., Wheeler M.L., Cho H.C., Popescu N.I., Coggeshall K.M., Arditi M. (2016). Hexokinase Is an Innate Immune Receptor for the Detection of Bacterial Peptidoglycan. Cell.

[109] Lee S.U., Li C.F., Mortales C.L., Pawling J., Dennis J.W., Grigorian A., Demetriou M. (2019). Increasing cell permeability of N-acetylglucosamine via 6-acetylation enhances capacity to suppress T-helper 1 (TH1)/TH17 responses and autoimmunity. PLoS ONE.

[110] Allmeroth K., Hartman M.D., Purrio M., Mesaros A., Denzel M.S. (2022). Hexosamine pathway activation improves memory but does not extend lifespan in mice. Aging Cell..

[111] Sun R.C., Young L.E.A., Bruntz R.C., Markussen K.H., Zhou Z., Conroy L.R., Hawkinson T.R., Clarke H.A., Stanback A.E., Macedo J.K.A. (2021). Brain glycogen serves as a critical glucosamine cache required for protein glycosylation. Cell. Metab..

[112] Cabib E., Roberts R., Bowers B. (1982). Synthesis of the yeast cell wall and its regulation. Annu. Rev. Biochem..

[113] Gaderer R., Seidl-Seiboth V., de Vries R.P., Seiboth B., Kappel L. (2017). N-acetylglucosamine, the building block of chitin, inhibits growth of Neurospora crassa. Fungal Genet. Biol..

[114] Udenfriend S., Kodukula K. (1995). How glycosylphosphatidylinositol-anchored membrane proteins are made. Annu. Rev. Biochem..

[115] Hang H.C., Bertozzi C.R. (2005). The chemistry and biology of mucin-type O-linked glycosylation. Bioorg. Med. Chem..

[116] Sames D., Chen X.T., Danishefsky S.J. (1997). Convergent total synthesis of a tumour-associated mucin motif. Nature.

[117] Helenius A., Aebi M. (2004). Roles of N-linked glycans in the endoplasmic reticulum. Annu. Rev. Biochem..

[118] Ruiz-Canada C., Kelleher D.J., Gilmore R. (2009). Cotranslational and posttranslational N-glycosylation of polypeptides by distinct mammalian OST isoforms. Cell.

[119] Walter P., Ron D. (2011). The unfolded protein response: From stress pathway to homeostatic regulation. Science.

[120] Wang Z.V., Deng Y., Wang Q.A., Sun K., Scherer P.E. (2010). Identification and characterization of a promoter cassette conferring adipocyte-specific gene expression. Endocrinology.

[121] Wong M.Y., Chen K., Antonopoulos A., Kasper B.T., Dewal M.B., Taylor R.J., Whittaker C.A., Hein P.P., Dell A., Genereux J.C. (2018). XBP1s activation can globally remodel N-glycan structure distribution patterns. Proc. Natl. Acad. Sci. USA.

[122] Hong M., Luo S., Baumeister P., Huang J.M., Gogia R.K., Li M., Lee A.S. (2004). Underglycosylation of ATF6 as a novel sensing mechanism for activation of the unfolded protein response. J. Biol. Chem..

[123] Nikonorova I.A., Mirek E.T., Signore C.C., Goudie M.P., Wek R.C., Anthony T.G. (2018). Time-resolved analysis of amino acid stress identifies eIF2 phosphorylation as necessary to inhibit mTORC1 activity in liver. J. Biol. Chem..

[124] Denzel M.S., Storm N.J., Gutschmidt A., Baddi R., Hinze Y., Jarosch E., Sommer T., Hoppe T., Antebi A. (2014). Hexosamine pathway metabolites enhance protein quality control and prolong life. Cell.

[125] Horn M., Denzel S.I., Srinivasan B., Allmeroth K., Schiffer I., Karthikaisamy V., Miethe S., Breuer P., Antebi A., Denzel M.S. (2020). Hexosamine Pathway Activation Improves Protein Homeostasis through the Integrated Stress Response. iScience.

[126] Ciraku L., Esquea E.M., Reginato M.J. (2022). O-GlcNAcylation regulation of cellular signaling in cancer. Cell. Signal..

[127] Zhu Y., Hart G.W. (2021). Targeting O-GlcNAcylation to develop novel therapeutics. Mol. Asp. Med..

[128] Chatham J.C., Young M.E., Zhang J. (2021). Role of O-linked N-acetylglucosamine (O-GlcNAc) modification of proteins in diabetic cardiovascular complications. Curr. Opin. Pharm..

[129] Yang X., Qian K. (2017). Protein O-GlcNAcylation: Emerging mechanisms and functions. Nat. Rev. Mol. Cell. Biol..

[130] Hardiville S., Hart G.W. (2014). Nutrient regulation of signaling, transcription, and cell physiology by O-GlcNAcylation. Cell. Metab..

[131] Kreppel L.K., Hart G.W. (1999). Regulation of a cytosolic and nuclear O-GlcNAc transferase. Role of the tetratricopeptide repeats. J. Biol. Chem..

[132] Liu K., Paterson A.J., Chin E., Kudlow J.E. (2000). Glucose stimulates protein modification by O-linked GlcNAc in pancreatic β cells: Linkage of O-linked GlcNAc to β cell death. Proc. Natl. Acad. Sci. USA.

[133] Phoomak C., Vaeteewoottacharn K., Silsirivanit A., Saengboonmee C., Seubwai W., Sawanyawisuth K., Wongkham C., Wongkham S. (2017). High glucose levels boost the aggressiveness of highly metastatic cholangiocarcinoma cells via O-GlcNAcylation. Sci. Rep..

[134] Cheung W.D., Hart G.W. (2008). AMP-activated protein kinase and p38 MAPK activate O-GlcNAcylation of neuronal proteins during glucose deprivation. J. Biol. Chem..

[135] Taylor R.P., Geisler T.S., Chambers J.H., McClain D.A. (2009). Up-regulation of O-GlcNAc transferase with glucose deprivation in HepG2 cells is mediated by decreased hexosamine pathway flux. J. Biol. Chem..

[136] Taylor R.P., Parker G.J., Hazel M.W., Soesanto Y., Fuller W., Yazzie M.J., McClain D.A. (2008). Glucose deprivation stimulates O-GlcNAc modification of proteins through up-regulation of O-linked N-acetylglucosaminyltransferase. J. Biol. Chem..

[137] Petrus P., Lecoutre S., Dollet L., Wiel C., Sulen A., Gao H., Tavira B., Laurencikiene J., Rooyackers O., Checa A. (2020). Glutamine Links Obesity to Inflammation in Human White Adipose Tissue. Cell. Metab..

[138] Yi W., Clark P.M., Mason D.E., Keenan M.C., Hill C., Goddard W.A., Peters E.C., Driggers E.M., Hsieh-Wilson L.C. (2012). Phosphofructokinase 1 glycosylation regulates cell growth and metabolism. Science.

[139] Wang Y., Liu J., Jin X., Zhang D., Li D., Hao F., Feng Y., Gu S., Meng F., Tian M. (2017). O-GlcNAcylation destabilizes the active tetrameric PKM2 to promote the Warburg effect. Proc. Natl. Acad. Sci. USA.

[140] Tan W., Jiang P., Zhang W., Hu Z., Lin S., Chen L., Li Y., Peng C., Li Z., Sun A. (2021). Posttranscriptional regulation of de novo lipogenesis by glucose-induced O-GlcNAcylation. Mol. Cell..

[141] Hu C.M., Tien S.C., Hsieh P.K., Jeng Y.M., Chang M.C., Chang Y.T., Chen Y.J., Chen Y.J., Lee E.Y.P., Lee W.H. (2019). High Glucose Triggers Nucleotide Imbalance through O-GlcNAcylation of Key Enzymes and Induces KRAS Mutation in Pancreatic Cells. Cell. Metab..

[142] Kim K., Yoo H.C., Kim B.G., Kim S., Sung Y., Yoon I., Yu Y.C., Park S.J., Kim J.H., Myung K. (2022). O-GlcNAc modification of leucyl-tRNA synthetase 1 integrates leucine and glucose availability to regulate mTORC1 and the metabolic fate of leucine. Nat. Commun..

[143] Chou T.Y., Hart G.W., Dang C.V. (1995). c-Myc is glycosylated at threonine 58, a known phosphorylation site and a mutational hot spot in lymphomas. J. Biol. Chem..

[144] Kamemura K., Hayes B.K., Comer F.I., Hart G.W. (2002). Dynamic interplay between O-glycosylation and O-phosphorylation of nucleocytoplasmic proteins: Alternative glycosylation/phosphorylation of THR-58, a known mutational hot spot of c-Myc in lymphomas, is regulated by mitogens. J. Biol. Chem..

[145] Itkonen H.M., Minner S., Guldvik I.J., Sandmann M.J., Tsourlakis M.C., Berge V., Svindland A., Schlomm T., Mills I.G. (2013). O-GlcNAc transferase integrates metabolic pathways to regulate the stability of c-MYC in human prostate cancer cells. Cancer Res..

[146] Lee D.H., Kwon N.E., Lee W.J., Lee M.S., Kim D.J., Kim J.H., Park S.K. (2020). Increased O-GlcNAcylation of c-Myc Promotes Pre-B Cell Proliferation. Cells.

[147] Housley M.P., Rodgers J.T., Udeshi N.D., Kelly T.J., Shabanowitz J., Hunt D.F., Puigserver P., Hart G.W. (2008). O-GlcNAc regulates FoxO activation in response to glucose. J. Biol. Chem..

[148] Wang S., Huang X., Sun D., Xin X., Pan Q., Peng S., Liang Z., Luo C., Yang Y., Jiang H. (2012). Extensive crosstalk between O-GlcNAcylation and phosphorylation regulates Akt signaling. PLoS ONE.

[149] Heath J.M., Sun Y., Yuan K., Bradley W.E., Litovsky S., Dell’Italia L.J., Chatham J.C., Wu H., Chen Y. (2014). Activation of AKT by O-linked N-acetylglucosamine induces vascular calcification in diabetes mellitus. Circ. Res..

[150] Sodi V.L., Khaku S., Krutilina R., Schwab L.P., Vocadlo D.J., Seagroves T.N., Reginato M.J. (2015). mTOR/MYC Axis Regulates O-GlcNAc Transferase Expression and O-GlcNAcylation in Breast Cancer. Mol. Cancer Res..

[151] Zhu Y., Hart G.W. (2021). Nutrient regulation of the flow of genetic information by O-GlcNAcylation. Biochem. Soc. Trans..

[152] Jia C., Li H., Fu D., Lan Y. (2020). GFAT1/HBP/O-GlcNAcylation Axis Regulates β-Catenin Activity to Promote Pancreatic Cancer Aggressiveness. BioMed Res. Int..

[153] Simpson N.E., Tryndyak V.P., Beland F.A., Pogribny I.P. (2012). An in vitro investigation of metabolically sensitive biomarkers in breast cancer progression. Breast Cancer Res. Treat..

[154] Vasconcelos-Dos-Santos A., Loponte H.F., Mantuano N.R., Oliveira I.A., de Paula I.F., Teixeira L.K., de-Freitas-Junior J.C., Gondim K.C., Heise N., Mohana-Borges R. (2017). Hyperglycemia exacerbates colon cancer malignancy through hexosamine biosynthetic pathway. Oncogenesis.

[155] Kim J., Lee H.M., Cai F., Ko B., Yang C., Lieu E.L., Muhammad N., Rhyne S., Li K., Haloul M. (2020). The hexosamine biosynthesis pathway is a targetable liability in KRAS/LKB1 mutant lung cancer. Nat. Metab..

[156] Ying H., Kimmelman A.C., Lyssiotis C.A., Hua S., Chu G.C., Fletcher-Sananikone E., Locasale J.W., Son J., Zhang H., Coloff J.L. (2012). Oncogenic Kras maintains pancreatic tumors through regulation of anabolic glucose metabolism. Cell.

[157] Guillaumond F., Leca J., Olivares O., Lavaut M.N., Vidal N., Berthezene P., Dusetti N.J., Loncle C., Calvo E., Turrini O. (2013). Strengthened glycolysis under hypoxia supports tumor symbiosis and hexosamine biosynthesis in pancreatic adenocarcinoma. Proc. Natl. Acad. Sci. USA.

[158] Chanmee T., Ontong P., Izumikawa T., Higashide M., Mochizuki N., Chokchaitaweesuk C., Khansai M., Nakajima K., Kakizaki I., Kongtawelert P. (2016). Hyaluronan Production Regulates Metabolic and Cancer Stem-like Properties of Breast Cancer Cells via Hexosamine Biosynthetic Pathway-coupled HIF-1 Signaling. J. Biol. Chem..

[159] Sharma N.S., Gupta V.K., Garrido V.T., Hadad R., Durden B.C., Kesh K., Giri B., Ferrantella A., Dudeja V., Saluja A. (2020). Targeting tumor-intrinsic hexosamine biosynthesis sensitizes pancreatic cancer to anti-PD1 therapy. J. Clin. Investig..

[160] Liu W., Jiang K., Wang J., Mei T., Zhao M., Huang D. (2021). Upregulation of GNPNAT1 Predicts Poor Prognosis and Correlates With Immune Infiltration in Lung Adenocarcinoma. Front. Mol. Biosci..

[161] Feng Y., Li N., Ren Y. (2022). GNPNAT1 Predicts Poor Prognosis and Cancer Development in Non-Small Cell Lung Cancer. Cancer Manag. Res..

[162] Zhu P., Gu S., Huang H., Zhong C., Liu Z., Zhang X., Wang W., Xie S., Wu K., Lu T. (2021). Upregulation of glucosamine-phosphate N-acetyltransferase 1 is a promising diagnostic and predictive indicator for poor survival in patients with lung adenocarcinoma. Oncol. Lett..

[163] Kaushik A.K., Shojaie A., Panzitt K., Sonavane R., Venghatakrishnan H., Manikkam M., Zaslavsky A., Putluri V., Vasu V.T., Zhang Y. (2016). Inhibition of the hexosamine biosynthetic pathway promotes castration-resistant prostate cancer. Nat. Commun..

[164] Lee H., Cai F., Kelekar N., Velupally N.K., Kim J. (2022). Targeting PGM3 as a Novel Therapeutic Strategy in KRAS/LKB1 Co-Mutant Lung Cancer. Cells.

[165] Ricciardiello F., Votta G., Palorini R., Raccagni I., Brunelli L., Paiotta A., Tinelli F., D’Orazio G., Valtorta S., De Gioia L. (2018). Inhibition of the Hexosamine Biosynthetic Pathway by targeting PGM3 causes breast cancer growth arrest and apoptosis. Cell. Death Dis..

[166] Zhang N., Liu S., Xu J., Ning T., Xie S., Min L., Zhu S., Zhang S., Zhu S. (2022). PGM3 regulates β-catenin activity to promote colorectal cancer cell progression. Exp. Biol. Med. (Maywood).

[167] Itkonen H.M., Engedal N., Babaie E., Luhr M., Guldvik I.J., Minner S., Hohloch J., Tsourlakis M.C., Schlomm T., Mills I.G. (2015). UAP1 is overexpressed in prostate cancer and is protective against inhibitors of N-linked glycosylation. Oncogene.

[168] Munkley J., Vodak D., Livermore K.E., James K., Wilson B.T., Knight B., McCullagh P., McGrath J., Crundwell M., Harries L.W. (2016). Glycosylation is an Androgen-Regulated Process Essential for Prostate Cancer Cell Viability. EBioMedicine.

[169] Puttamallesh V.N., Deb B., Gondkar K., Jain A., Nair B., Pandey A., Chatterjee A., Gowda H., Kumar P. (2020). Quantitative Proteomics of Urinary Bladder Cancer Cell Lines Identify UAP1 as a Potential Therapeutic Target. Genes.

[170] Su Z., Wang C., Pan R., Li H., Chen J., Tan J., Tian X., Lin T., Shen J. (2022). The hexosamine biosynthesis pathway-related gene signature correlates with immune infiltration and predicts prognosis of patients with osteosarcoma. Front. Immunol..

[171] Wang X., Chen X., Liu H. (2020). Expression and Bioinformatics-Based Functional Analysis of UAP1 in Lung Adenocarcinoma. Cancer Manag. Res..

[172] Gao S., Miao Y., Liu Y., Liu X., Fan X., Lin Y., Qian P., Zhou J., Dai Y., Xia L. (2019). Reciprocal Regulation Between O-GlcNAcylation and β-Catenin Facilitates Cell Viability and Inhibits Apoptosis in Liver Cancer. DNA Cell. Biol..

[173] Lai C.Y., Liu H., Tin K.X., Huang Y., Yeh K.H., Peng H.W., Chen H.D., He J.Y., Chiang Y.J., Liu C.S. (2019). Identification of UAP1L1 as a critical factor for protein O-GlcNAcylation and cell proliferation in human hepatoma cells. Oncogene.

[174] Wu X.C., Yu Y.Z., Zuo Y.Z., Song X.L., Zhou Z.E., Xiao Y., Luo D.S., Yan W.G., Zhao S.C. (2022). Identification of UAP1L1 as a critical factor for prostate cancer and underlying molecular mechanism in tumorigenicity. J. Transl. Med..

[175] Hill V.K., Ricketts C., Bieche I., Vacher S., Gentle D., Lewis C., Maher E.R., Latif F. (2011). Genome-wide DNA methylation profiling of CpG islands in breast cancer identifies novel genes associated with tumorigenicity. Cancer Res..

[176] Yang Y., Vankayalapati H., Tang M., Zheng Y., Li Y., Ma C., Lai K. (2020). Discovery of Novel Inhibitors Targeting Multi-UDP-hexose Pyrophosphorylases as Anticancer Agents. Molecules.

[177] Raimi O.G., Hurtado-Guerrero R., Borodkin V., Ferenbach A., Urbaniak M.D., Ferguson M.A.J., van Aalten D.M.F. (2020). A mechanism-inspired UDP-N-acetylglucosamine pyrophosphorylase inhibitor. RSC Chem. Biol..

[178] Lau K.S., Partridge E.A., Grigorian A., Silvescu C.I., Reinhold V.N., Demetriou M., Dennis J.W. (2007). Complex N-glycan number and degree of branching cooperate to regulate cell proliferation and differentiation. Cell.

[179] Dragic H., Barthelaix A., Duret C., Le Goupil S., Laprade H., Martin S., Brugiere S., Coute Y., Machon C., Guitton J. (2022). The hexosamine pathway and coat complex II promote malignant adaptation to nutrient scarcity. Life Sci. Alliance.

[180] Pinho S.S., Reis C.A. (2015). Glycosylation in cancer: Mechanisms and clinical implications. Nat. Rev. Cancer.

[181] Gu Y., Mi W., Ge Y., Liu H., Fan Q., Han C., Yang J., Han F., Lu X., Yu W. (2010). GlcNAcylation plays an essential role in breast cancer metastasis. Cancer Res..

[182] Caldwell S.A., Jackson S.R., Shahriari K.S., Lynch T.P., Sethi G., Walker S., Vosseller K., Reginato M.J. (2010). Nutrient sensor O-GlcNAc transferase regulates breast cancer tumorigenesis through targeting of the oncogenic transcription factor FoxM1. Oncogene.

[183] Lynch T.P., Ferrer C.M., Jackson S.R., Shahriari K.S., Vosseller K., Reginato M.J. (2012). Critical role of O-Linked β-N-acetylglucosamine transferase in prostate cancer invasion, angiogenesis, and metastasis. J. Biol. Chem..

[184] Ferrer C.M., Sodi V.L., Reginato M.J. (2016). O-GlcNAcylation in Cancer Biology: Linking Metabolism and Signaling. J. Mol. Biol..

[185] Fardini Y., Dehennaut V., Lefebvre T., Issad T. (2013). O-GlcNAcylation: A New Cancer Hallmark?. Front. Endocrinol..

[186] Guo H., Zhang B., Nairn A.V., Nagy T., Moremen K.W., Buckhaults P., Pierce M. (2017). O-Linked N-Acetylglucosamine (O-GlcNAc) Expression Levels Epigenetically Regulate Colon Cancer Tumorigenesis by Affecting the Cancer Stem Cell Compartment via Modulating Expression of Transcriptional Factor MYBL1. J. Biol. Chem..

[187] Peng C., Zhu Y., Zhang W., Liao Q., Chen Y., Zhao X., Guo Q., Shen P., Zhen B., Qian X. (2017). Regulation of the Hippo-YAP Pathway by Glucose Sensor O-GlcNAcylation. Mol. Cell..

[188] Singh J.P., Qian K., Lee J.S., Zhou J., Han X., Zhang B., Ong Q., Ni W., Jiang M., Ruan H.B. (2020). O-GlcNAcase targets pyruvate kinase M2 to regulate tumor growth. Oncogene.

[189] Harosh-Davidovich S.B., Khalaila I. (2018). O-GlcNAcylation affects β-catenin and E-cadherin expression, cell motility and tumorigenicity of colorectal cancer. Exp. Cell. Res..

[190] Hanover J.A., Krause M.W., Love D.C. (2012). Bittersweet memories: Linking metabolism to epigenetics through O-GlcNAcylation. Nat. Rev. Mol. Cell. Biol..

[191] Sun L., Lv S., Song T. (2021). O-GlcNAcylation links oncogenic signals and cancer epigenetics. Discov. Oncol..

[192] Marshall S., Bacote V., Traxinger R.R. (1991). Complete inhibition of glucose-induced desensitization of the glucose transport system by inhibitors of mRNA synthesis. Evidence for rapid turnover of glutamine:fructose-6-phosphate amidotransferase. J. Biol. Chem..

[193] McClain D.A., Crook E.D. (1996). Hexosamines and insulin resistance. Diabetes.

[194] Rossetti L., Hawkins M., Chen W., Gindi J., Barzilai N. (1995). In vivo glucosamine infusion induces insulin resistance in normoglycemic but not in hyperglycemic conscious rats. J. Clin. Investig..

[195] Buse M.G., Robinson K.A., Gettys T.W., McMahon E.G., Gulve E.A. (1997). Increased activity of the hexosamine synthesis pathway in muscles of insulin-resistant ob/ob mice. Am. J. Physiol..

[196] Robinson K.A., Weinstein M.L., Lindenmayer G.E., Buse M.G. (1995). Effects of diabetes and hyperglycemia on the hexosamine synthesis pathway in rat muscle and liver. Diabetes.

[197] Akimoto Y., Hart G.W., Wells L., Vosseller K., Yamamoto K., Munetomo E., Ohara-Imaizumi M., Nishiwaki C., Nagamatsu S., Hirano H. (2007). Elevation of the post-translational modification of proteins by O-linked N-acetylglucosamine leads to deterioration of the glucose-stimulated insulin secretion in the pancreas of diabetic Goto-Kakizaki rats. Glycobiology.

[198] Yki-Jarvinen H., Daniels M.C., Virkamaki A., Makimattila S., DeFronzo R.A., McClain D. (1996). Increased glutamine:fructose-6-phosphate amidotransferase activity in skeletal muscle of patients with NIDDM. Diabetes.

[199] Srinivasan V., Sandhya N., Sampathkumar R., Farooq S., Mohan V., Balasubramanyam M. (2007). Glutamine fructose-6-phosphate amidotransferase (GFAT) gene expression and activity in patients with type 2 diabetes: Inter-relationships with hyperglycaemia and oxidative stress. Clin. Biochem..

[200] Hebert L.F., Daniels M.C., Zhou J., Crook E.D., Turner R.L., Simmons S.T., Neidigh J.L., Zhu J.S., Baron A.D., McClain D.A. (1996). Overexpression of glutamine:fructose-6-phosphate amidotransferase in transgenic mice leads to insulin resistance. J. Clin. Investig..

[201] Tang J., Neidigh J.L., Cooksey R.C., McClain D.A. (2000). Transgenic mice with increased hexosamine flux specifically targeted to β-cells exhibit hyperinsulinemia and peripheral insulin resistance. Diabetes.

[202] Veerababu G., Tang J., Hoffman R.T., Daniels M.C., Hebert L.F., Crook E.D., Cooksey R.C., McClain D.A. (2000). Overexpression of glutamine: Fructose-6-phosphate amidotransferase in the liver of transgenic mice results in enhanced glycogen storage, hyperlipidemia, obesity, and impaired glucose tolerance. Diabetes.

[203] Sage A.T., Walter L.A., Shi Y., Khan M.I., Kaneto H., Capretta A., Werstuck G.H. (2010). Hexosamine biosynthesis pathway flux promotes endoplasmic reticulum stress, lipid accumulation, and inflammatory gene expression in hepatic cells. Am. J. Physiol. Endocrinol. Metab..

[204] Wu R., Zhang Q.H., Lu Y.J., Ren K., Yi G.H. (2015). Involvement of the IRE1alpha-XBP1 pathway and XBP1s-dependent transcriptional reprogramming in metabolic diseases. DNA Cell. Biol..

[205] Ozcan U., Cao Q., Yilmaz E., Lee A.H., Iwakoshi N.N., Ozdelen E., Tuncman G., Gorgun C., Glimcher L.H., Hotamisligil G.S. (2004). Endoplasmic reticulum stress links obesity, insulin action, and type 2 diabetes. Science.

[206] Yilmaz E. (2017). Endoplasmic Reticulum Stress and Obesity. Adv. Exp. Med. Biol..

[207] Pan H.T., Xiong Y.M., Zhu H.D., Shi X.L., Yu B., Ding H.G., Xu R.J., Ding J.L., Zhang T., Zhang J. (2022). Proteomics and bioinformatics analysis of cardiovascular related proteins in offspring exposed to gestational diabetes mellitus. Front. Cardiovasc. Med..

[208] Marshall S., Garvey W.T., Traxinger R.R. (1991). New insights into the metabolic regulation of insulin action and insulin resistance: Role of glucose and amino acids. FASEB J..

[209] Cooksey R.C., Hebert L.F., Zhu J.H., Wofford P., Garvey W.T., McClain D.A. (1999). Mechanism of hexosamine-induced insulin resistance in transgenic mice overexpressing glutamine:fructose-6-phosphate amidotransferase: Decreased glucose transporter GLUT4 translocation and reversal by treatment with thiazolidinedione. Endocrinology.

[210] Baron A.D., Zhu J.S., Zhu J.H., Weldon H., Maianu L., Garvey W.T. (1995). Glucosamine induces insulin resistance in vivo by affecting GLUT 4 translocation in skeletal muscle. Implications for glucose toxicity. J. Clin. Investig..

[211] Ohtsubo K., Takamatsu S., Minowa M.T., Yoshida A., Takeuchi M., Marth J.D. (2005). Dietary and genetic control of glucose transporter 2 glycosylation promotes insulin secretion in suppressing diabetes. Cell.

[212] Ohtsubo K., Chen M.Z., Olefsky J.M., Marth J.D. (2011). Pathway to diabetes through attenuation of pancreatic β cell glycosylation and glucose transport. Nat. Med..

[213] Haga Y., Ishii K., Suzuki T. (2011). N-glycosylation is critical for the stability and intracellular trafficking of glucose transporter GLUT4. J. Biol. Chem..

[214] Nelson B.A., Robinson K.A., Buse M.G. (2002). Insulin acutely regulates Munc18-c subcellular trafficking: Altered response in insulin-resistant 3T3-L1 adipocytes. J. Biol. Chem..

[215] Chen G., Liu P., Thurmond D.C., Elmendorf J.S. (2003). Glucosamine-induced insulin resistance is coupled to O-linked glycosylation of Munc18c. FEBS Lett..

[216] Alejandro E.U., Bozadjieva N., Kumusoglu D., Abdulhamid S., Levine H., Haataja L., Vadrevu S., Satin L.S., Arvan P., Bernal-Mizrachi E. (2015). Disruption of O-linked N-Acetylglucosamine Signaling Induces ER Stress and β Cell Failure. Cell. Rep..

[217] Jo S., Pritchard S., Wong A., Avula N., Essawy A., Hanover J., Alejandro E.U. (2022). Pancreatic β-cell hyper-O-GlcNAcylation leads to impaired glucose homeostasis in vivo. Front. Endocrinol..

[218] Ma J., Hart G.W. (2013). Protein O-GlcNAcylation in diabetes and diabetic complications. Expert. Rev. Proteom..

[219] Whelan S.A., Dias W.B., Thiruneelakantapillai L., Lane M.D., Hart G.W. (2010). Regulation of insulin receptor substrate 1 (IRS-1)/AKT kinase-mediated insulin signaling by O-Linked β-N-acetylglucosamine in 3T3-L1 adipocytes. J. Biol. Chem..

[220] Salguero A.L., Chen M., Balana A.T., Chu N., Jiang H., Palanski B.A., Bae H., Wright K.M., Nathan S., Zhu H. (2022). Multifaceted Regulation of Akt by Diverse C-terminal Post-translational Modifications. ACS Chem. Biol..

[221] de Jesus T.J., Tomalka J.A., Centore J.T., Staback Rodriguez F.D., Agarwal R.A., Liu A.R., Kern T.S., Ramakrishnan P. (2021). Negative regulation of FOXP3 expression by c-Rel O-GlcNAcylation. Glycobiology.

[222] Dentin R., Hedrick S., Xie J., Yates J., Montminy M. (2008). Hepatic glucose sensing via the CREB coactivator CRTC2. Science.

[223] Gonzalez-Rellan M.J., Fondevila M.F., Fernandez U., Rodriguez A., Varela-Rey M., Veyrat-Durebex C., Seoane S., Bernardo G., Lopitz-Otsoa F., Fernandez-Ramos D. (2021). O-GlcNAcylated p53 in the liver modulates hepatic glucose production. Nat. Commun..

[224] Baldini S.F., Steenackers A., Olivier-Van Stichelen S., Mir A.M., Mortuaire M., Lefebvre T., Guinez C. (2016). Glucokinase expression is regulated by glucose through O-GlcNAc glycosylation. Biochem. Biophys. Res. Commun..

[225] Robinson K.A., Ball L.E., Buse M.G. (2007). Reduction of O-GlcNAc protein modification does not prevent insulin resistance in 3T3-L1 adipocytes. Am. J. Physiol. Endocrinol. Metab..

[226] Springhorn C., Matsha T.E., Erasmus R.T., Essop M.F. (2012). Exploring leukocyte O-GlcNAcylation as a novel diagnostic tool for the earlier detection of type 2 diabetes mellitus. J. Clin. Endocrinol. Metab..

[227] Fallahi M., Jamee M., Enayat J., Abdollahimajd F., Mesdaghi M., Khoddami M., Segarra-Roca A., Frohne A., Dmytrus J., Keramatipour M. (2022). Novel PGM3 mutation in two siblings with combined immunodeficiency and childhood bullous pemphigoid: A case report and review of the literature. Allergy Asthma Clin. Immunol..

[228] Fusaro M., Vincent A., Castelle M., Rosain J., Fournier B., Veiga-da-Cunha M., Kentache T., Serre J., Fallet-Bianco C., Delezoide A.L. (2021). Two Novel Homozygous Mutations in Phosphoglucomutase 3 Leading to Severe Combined Immunodeficiency, Skeletal Dysplasia, and Malformations. J. Clin. Immunol..

[229] Lundin K.E., Wang Q., Hamasy A., Marits P., Uzunel M., Wirta V., Wikstrom A.C., Fasth A., Ekwall O., Smith C.I.E. (2018). Eleven percent intact PGM3 in a severely immunodeficient patient with a novel splice-site mutation, a case report. BMC Pediatr..

[230] Bernth-Jensen J.M., Holm M., Christiansen M. (2016). Neonatal-onset T^−^B^−^NK^+^ severe combined immunodeficiency and neutropenia caused by mutated phosphoglucomutase 3. J. Allergy Clin. Immunol..

[231] Yang L., Fliegauf M., Grimbacher B. (2014). Hyper-IgE syndromes: Reviewing PGM3 deficiency. Curr. Opin. Pediatr..

[232] Zhou R.W., Mkhikian H., Grigorian A., Hong A., Chen D., Arakelyan A., Demetriou M. (2014). N-glycosylation bidirectionally extends the boundaries of thymocyte positive selection by decoupling Lck from Ca^2+^ signaling. Nat. Immunol..

[233] Demetriou M., Granovsky M., Quaggin S., Dennis J.W. (2001). Negative regulation of T-cell activation and autoimmunity by Mgat5 N-glycosylation. Nature.

[234] Dias A.M., Correia A., Pereira M.S., Almeida C.R., Alves I., Pinto V., Catarino T.A., Mendes N., Leander M., Oliva-Teles M.T. (2018). Metabolic control of T cell immune response through glycans in inflammatory bowel disease. Proc. Natl. Acad. Sci. USA.

[235] Brandt A.U., Sy M., Bellmann-Strobl J., Newton B.L., Pawling J., Zimmermann H.G., Yu Z., Chien C., Dorr J., Wuerfel J.T. (2021). Association of a Marker of N-Acetylglucosamine With Progressive Multiple Sclerosis and Neurodegeneration. JAMA Neurol..

[236] Mortales C.L., Lee S.U., Manousadjian A., Hayama K.L., Demetriou M. (2020). N-Glycan Branching Decouples B Cell Innate and Adaptive Immunity to Control Inflammatory Demyelination. iScience.

[237] Mkhikian H., Hayama K.L., Khachikyan K., Li C., Zhou R.W., Pawling J., Klaus S., Tran P.Q.N., Ly K.M., Gong A.D. (2022). Age-associated impairment of T cell immunity is linked to sex-dimorphic elevation of N-glycan branching. Nat. Aging.

[238] Ma L., Rudert W.A., Harnaha J., Wright M., Machen J., Lakomy R., Qian S., Lu L., Robbins P.D., Trucco M. (2002). Immunosuppressive effects of glucosamine. J. Biol. Chem..

[239] Chen N.H., Cheong K.A., Kim C.H., Noh M., Lee A.Y. (2013). Glucosamine induces activated T cell apoptosis through reduced T cell receptor. Scand. J. Immunol..

[240] Zhang G.X., Yu S., Gran B., Rostami A. (2005). Glucosamine abrogates the acute phase of experimental autoimmune encephalomyelitis by induction of Th2 response. J. Immunol..

[241] Chien M.W., Lin M.H., Huang S.H., Fu S.H., Hsu C.Y., Yen B.L., Chen J.T., Chang D.M., Sytwu H.K. (2015). Glucosamine Modulates T Cell Differentiation through Down-regulating N-Linked Glycosylation of CD25. J. Biol. Chem..

[242] Kim C.H., Cheong K.A., Park C.D., Lee A.Y. (2011). Glucosamine improved atopic dermatitis-like skin lesions in NC/Nga mice by inhibition of Th2 cell development. Scand. J. Immunol..

[243] Kim C.H., Kim J.Y., Lee A.Y. (2015). Therapeutic and immunomodulatory effects of glucosamine in combination with low-dose cyclosporine a in a murine model of imiquimod-induced psoriasis. Eur. J. Pharmacol..

[244] Swamy M., Pathak S., Grzes K.M., Damerow S., Sinclair L.V., van Aalten D.M., Cantrell D.A. (2016). Glucose and glutamine fuel protein O-GlcNAcylation to control T cell self-renewal and malignancy. Nat. Immunol..

[245] Lund P.J., Elias J.E., Davis M.M. (2016). Global Analysis of O-GlcNAc Glycoproteins in Activated Human T Cells. J. Immunol..

[246] Abramowitz L.K., Harly C., Das A., Bhandoola A., Hanover J.A. (2019). Blocked O-GlcNAc cycling disrupts mouse hematopoeitic stem cell maintenance and early T cell development. Sci. Rep..

[247] Liu B., Salgado O.C., Singh S., Hippen K.L., Maynard J.C., Burlingame A.L., Ball L.E., Blazar B.R., Farrar M.A., Hogquist K.A. (2019). The lineage stability and suppressive program of regulatory T cells require protein O-GlcNAcylation. Nat. Commun..

[248] Golks A., Tran T.T., Goetschy J.F., Guerini D. (2007). Requirement for O-linked N-acetylglucosaminyltransferase in lymphocytes activation. EMBO J..

[249] Wu J.L., Chiang M.F., Hsu P.H., Tsai D.Y., Hung K.H., Wang Y.H., Angata T., Lin K.I. (2017). O-GlcNAcylation is required for B cell homeostasis and antibody responses. Nat. Commun..

[250] Wu J.L., Wu H.Y., Tsai D.Y., Chiang M.F., Chen Y.J., Gao S., Lin C.C., Lin C.H., Khoo K.H., Chen Y.J. (2016). Temporal regulation of Lsp1 O-GlcNAcylation and phosphorylation during apoptosis of activated B cells. Nat. Commun..

[251] Mereiter S., Balmana M., Campos D., Gomes J., Reis C.A. (2019). Glycosylation in the Era of Cancer-Targeted Therapy: Where Are We Heading?. Cancer Cell.

[252] Chen B., Liu W., Li Y., Ma B., Shang S., Tan Z. (2022). Impact of N-Linked Glycosylation on Therapeutic Proteins. Molecules.

[253] Jha A.K., Huang S.C., Sergushichev A., Lampropoulou V., Ivanova Y., Loginicheva E., Chmielewski K., Stewart K.M., Ashall J., Everts B. (2015). Network integration of parallel metabolic and transcriptional data reveals metabolic modules that regulate macrophage polarization. Immunity.

[254] Puchalska P., Huang X., Martin S.E., Han X., Patti G.J., Crawford P.A. (2018). Isotope Tracing Untargeted Metabolomics Reveals Macrophage Polarization-State-Specific Metabolic Coordination across Intracellular Compartments. iScience.

[255] Kumar A., Yarosz E.L., Andren A., Zhang L., Lyssiotis C.A., Chang C.H. (2022). NKT cells adopt a glutamine-addicted phenotype to regulate their homeostasis and function. Cell. Rep..

[256] Tran D.H., May H.I., Li Q., Luo X., Huang J., Zhang G., Niewold E., Wang X., Gillette T.G., Deng Y. (2020). Chronic activation of hexosamine biosynthesis in the heart triggers pathological cardiac remodeling. Nat. Commun..

[257] Rajamani U., Essop M.F. (2010). Hyperglycemia-mediated activation of the hexosamine biosynthetic pathway results in myocardial apoptosis. Am. J. Physiol. Cell. Physiol..

[258] Senderek J., Muller J.S., Dusl M., Strom T.M., Guergueltcheva V., Diepolder I., Laval S.H., Maxwell S., Cossins J., Krause S. (2011). Hexosamine biosynthetic pathway mutations cause neuromuscular transmission defect. Am. J. Hum. Genet..

[259] Guergueltcheva V., Muller J.S., Dusl M., Senderek J., Oldfors A., Lindbergh C., Maxwell S., Colomer J., Mallebrera C.J., Nascimento A. (2012). Congenital myasthenic syndrome with tubular aggregates caused by GFPT1 mutations. J. Neurol..

[260] Selcen D., Shen X.M., Milone M., Brengman J., Ohno K., Deymeer F., Finkel R., Rowin J., Engel A.G. (2013). GFPT1-myasthenia: Clinical, structural, and electrophysiologic heterogeneity. Neurology.

[261] Huh S.Y., Kim H.S., Jang H.J., Park Y.E., Kim D.S. (2012). Limb-girdle myasthenia with tubular aggregates associated with novel GFPT1 mutations. Muscle Nerve.

[262] Maselli R.A., Arredondo J., Nguyen J., Lara M., Ng F., Ngo M., Pham J.M., Yi Q., Stajich J.M., McDonald K. (2014). Exome sequencing detection of two untranslated GFPT1 mutations in a family with limb-girdle myasthenia. Clin. Genet..

[263] Chen Q., Muller J.S., Pang P.C., Laval S.H., Haslam S.M., Lochmuller H., Dell A. (2015). Global N-linked Glycosylation is Not Significantly Impaired in Myoblasts in Congenital Myasthenic Syndromes Caused by Defective Glutamine-Fructose-6-Phosphate Transaminase 1 (GFPT1). Biomolecules.

[264] Zoltowska K., Webster R., Finlayson S., Maxwell S., Cossins J., Muller J., Lochmuller H., Beeson D. (2013). Mutations in GFPT1 that underlie limb-girdle congenital myasthenic syndrome result in reduced cell-surface expression of muscle AChR. Hum. Mol. Genet..

[265] Gehle V.M., Walcott E.C., Nishizaki T., Sumikawa K. (1997). N-glycosylation at the conserved sites ensures the expression of properly folded functional ACh receptors. Brain Res. Mol. Brain Res..

[266] Issop Y., Hathazi D., Khan M.M., Rudolf R., Weis J., Spendiff S., Slater C.R., Roos A., Lochmuller H. (2018). GFPT1 deficiency in muscle leads to myasthenia and myopathy in mice. Hum. Mol. Genet..

[267] Ain N.U., Baroncelli M., Costantini A., Ishaq T., Taylan F., Nilsson O., Makitie O., Naz S. (2021). Novel form of rhizomelic skeletal dysplasia associated with a homozygous variant in GNPNAT1. J. Med. Genet..

[268] Elhossini R.M., Ahmed H.A., Otaify G., Ghorab R.M., Amr K., Aglan M. (2022). A novel variant in GNPNAT1 gene causing a spondylo-epi-metaphyseal dysplasia resembling PGM3-Desbuquois like dysplasia. Am. J. Med. Genet. A.

[269] Stray-Pedersen A., Backe P.H., Sorte H.S., Morkrid L., Chokshi N.Y., Erichsen H.C., Gambin T., Elgstoen K.B., Bjoras M., Wlodarski M.W. (2014). PGM3 mutations cause a congenital disorder of glycosylation with severe immunodeficiency and skeletal dysplasia. Am. J. Hum. Genet..

[270] Zhang Y., Yu X., Ichikawa M., Lyons J.J., Datta S., Lamborn I.T., Jing H., Kim E.S., Biancalana M., Wolfe L.A. (2014). Autosomal recessive phosphoglucomutase 3 (PGM3) mutations link glycosylation defects to atopy, immune deficiency, autoimmunity, and neurocognitive impairment. J. Allergy Clin. Immunol..

[271] Chen X., Raimi O.G., Ferenbach A.T., van Aalten D.M.F. (2021). A missense mutation in a patient with developmental delay affects the activity and structure of the hexosamine biosynthetic pathway enzyme AGX1. FEBS Lett..

[272] Chang I.J., He M., Lam C.T. (2018). Congenital disorders of glycosylation. Ann. Transl. Med..

[273] Lemberg K.M., Vornov J.J., Rais R., Slusher B.S. (2018). We’re Not “DON” Yet: Optimal Dosing and Prodrug Delivery of 6-Diazo-5-oxo-L-norleucine. Mol. Cancer Ther..

[274] Bartz Q.R., Elder C.C., Frohardt R.P., Fusari S.A., Haskell T.H., Johannessen D.W., Ryder A. (1954). Isolation and characterization of azaserine. Nature.

[275] Ehrlich J., Anderson L.E., Coffey G.L., Hillegas A.B., Knudsen M.P., Koepsell H.J., Kohberger D.L., Oyaas J.E. (1954). Antibiotic studies of azaserine. Nature.

[276] Catane R., Von Hoff D.D., Glaubiger D.L., Muggia F.M. (1979). Azaserine, DON, and azotomycin: Three diazo analogs of L-glutamine with clinical antitumor activity. Cancer Treat. Rep..

[277] Livingston R.B., Venditti J.M., Cooney D.A., Carter S.K. (1970). Glutamine antagonists in chemotherapy. Adv. Pharm. Chemother..

[278] Tarnowski G.S., Stock C.C. (1957). Effects of combinations of azaserine and of 6-diazo-5-oxo-L-norleucine with purine analogs and other antimetabolites on the growth of two mouse mammary carcinomas. Cancer Res..

[279] Ovejera A.A., Houchens D.P., Catane R., Sheridan M.A., Muggia F.M. (1979). Efficacy of 6-diazo-5-oxo-L-norleucine and N-[N-γ-glutamyl-6-diazo-5-oxo-norleucinyl]-6-diazo-5-oxo-norleucine against experimental tumors in conventional and nude mice. Cancer Res..

[280] Earhart R.H., Amato D.J., Chang A.Y., Borden E.C., Shiraki M., Dowd M.E., Comis R.L., Davis T.E., Smith T.J. (1990). Phase II trial of 6-diazo-5-oxo-L-norleucine versus aclacinomycin-A in advanced sarcomas and mesotheliomas. Investig. New. Drugs.

[281] Lynch G., Kemeny N., Casper E. (1982). Phase II evaluation of DON (6-diazo-5-oxo-L-norleucine) in patients with advanced colorectal carcinoma. Am. J. Clin. Oncol..

[282] Asthana A., Ramakrishnan P., Vicioso Y., Zhang K., Parameswaran R. (2018). Hexosamine Biosynthetic Pathway Inhibition Leads to AML Cell Differentiation and Cell Death. Mol. Cancer Ther..

[283] Chen W., Do K.C., Saxton B., Leng S., Filipczak P., Tessema M., Belinsky S.A., Lin Y. (2019). Inhibition of the hexosamine biosynthesis pathway potentiates cisplatin cytotoxicity by decreasing BiP expression in non-small-cell lung cancer cells. Mol. Carcinog..

[284] Walter L.A., Lin Y.H., Halbrook C.J., Chuh K.N., He L., Pedowitz N.J., Batt A.R., Brennan C.K., Stiles B.L., Lyssiotis C.A. (2020). Inhibiting the Hexosamine Biosynthetic Pathway Lowers O-GlcNAcylation Levels and Sensitizes Cancer to Environmental Stress. Biochemistry.

[285] Chen W., Saxton B., Tessema M., Belinsky S.A. (2021). Inhibition of GFAT1 in lung cancer cells destabilizes PD-L1 protein. Carcinogenesis.

[286] Rais R., Jancarik A., Tenora L., Nedelcovych M., Alt J., Englert J., Rojas C., Le A., Elgogary A., Tan J. (2016). Discovery of 6-Diazo-5-oxo-l-norleucine (DON) Prodrugs with Enhanced CSF Delivery in Monkeys: A Potential Treatment for Glioblastoma. J. Med. Chem..

[287] Ueki N., Lee S., Sampson N.S., Hayman M.J. (2013). Selective cancer targeting with prodrugs activated by histone deacetylases and a tumour-associated protease. Nat. Commun..

[288] Zhang Y., Li J., Huang Y., Chen Y., Luo Z., Huang H., West R.E., Nolin T.D., Wang Z., Li S. (2023). Improved antitumor activity against prostate cancer via synergistic targeting of Myc and GFAT-1. Theranostics.

[289] Vyas B., Silakari O., Bahia M.S., Singh B. (2013). Glutamine: Fructose-6-phosphate amidotransferase (GFAT): Homology modelling and designing of new inhibitors using pharmacophore and docking based hierarchical virtual screening protocol. SAR QSAR Env. Res..

[290] Ricciardiello F., Gang Y., Palorini R., Li Q., Giampa M., Zhao F., You L., La Ferla B., De Vitto H., Guan W. (2020). Hexosamine pathway inhibition overcomes pancreatic cancer resistance to gemcitabine through unfolded protein response and EGFR-Akt pathway modulation. Oncogene.

[291] Ricciardiello F., Bergamaschi L., De Vitto H., Gang Y., Zhang T., Palorini R., Chiaradonna F. (2021). Suppression of the HBP Function Increases Pancreatic Cancer Cell Sensitivity to a Pan-RAS Inhibitor. Cells.

[292] Miwa I., Mita Y., Murata T., Okuda J., Sugiura M., Hamada Y., Chiba T. (1994). Utility of 3-O-methyl-N-acetyl-D-glucosamine, an N-acetylglucosamine kinase inhibitor, for accurate assay of glucokinase in pancreatic islets and liver. Enzym. Protein.

[293] Zeitler R., Giannis A., Danneschewski S., Henk E., Henk T., Bauer C., Reutter W., Sandhoff K. (1992). Inhibition of N-acetylglucosamine kinase and N-acetylmannosamine kinase by 3-O-methyl-N-acetyl-D-glucosamine in vitro. Eur. J. Biochem..

[294] Heifetz A., Keenan R.W., Elbein A.D. (1979). Mechanism of action of tunicamycin on the UDP-GlcNAc:dolichyl-phosphate Glc-NAc-1-phosphate transferase. Biochemistry.

[295] Liu J., Marchase R.B., Chatham J.C. (2007). Glutamine-induced protection of isolated rat heart from ischemia/reperfusion injury is mediated via the hexosamine biosynthesis pathway and increased protein O-GlcNAc levels. J. Mol. Cell. Cardiol..

[296] Kaneto H., Xu G., Song K.H., Suzuma K., Bonner-Weir S., Sharma A., Weir G.C. (2001). Activation of the hexosamine pathway leads to deterioration of pancreatic β-cell function through the induction of oxidative stress. J. Biol. Chem..

[297] Ishino K., Kudo M., Peng W.X., Kure S., Kawahara K., Teduka K., Kawamoto Y., Kitamura T., Fujii T., Yamamoto T. (2018). 2-Deoxy-d-glucose increases GFAT1 phosphorylation resulting in endoplasmic reticulum-related apoptosis via disruption of protein N-glycosylation in pancreatic cancer cells. Biochem. Biophys. Res. Commun..

[298] DeBerardinis R.J., Cheng T. (2010). Q’s next: The diverse functions of glutamine in metabolism, cell biology and cancer. Oncogene.

[299] Engle D.D., Tiriac H., Rivera K.D., Pommier A., Whalen S., Oni T.E., Alagesan B., Lee E.J., Yao M.A., Lucito M.S. (2019). The glycan CA19-9 promotes pancreatitis and pancreatic cancer in mice. Science.

[300] Rudman N., Gornik O., Lauc G. (2019). Altered N-glycosylation profiles as potential biomarkers and drug targets in diabetes. FEBS Lett..

